# Laser Processing of Hard and Ultra-Hard Materials for Micro-Machining and Surface Engineering Applications

**DOI:** 10.3390/mi12080895

**Published:** 2021-07-28

**Authors:** Kafayat Eniola Hazzan, Manuela Pacella, Tian Long See

**Affiliations:** 1 Wolfson School of Mechanical, Electrical and Manufacturing Engineering, Loughborough University, Loughborough LE11 3TU, UK; K.Hazzan@lboro.ac.uk; 2 The Manufacturing Technology Centre (MTC) Ltd., Pilot Way, Ansty Park, Coventry CV7 9JU, UK; TianLong.See@the-mtc.org

**Keywords:** laser-based micromachining, laser processing, polycrystalline boron nitride, polycrystalline diamond, tungsten carbide, surface texturing, cutting tools

## Abstract

Polycrystalline diamonds, polycrystalline cubic boron nitrides and tungsten carbides are considered difficult to process due to their superior mechanical (hardness, toughness) and wear properties. This paper aims to review the recent progress in the use of lasers to texture hard and ultra-hard materials to a high and reproducible quality. The effect of wavelength, beam type, pulse duration, fluence, and scanning speed is extensively reviewed, and the resulting laser mechanisms, induced damage, surface integrity, and existing challenges discussed. The cutting performance of different textures in real applications is examined, and the key influence of texture size, texture geometry, area ratio, area density, orientation, and solid lubricants is highlighted. Pulsed laser ablation (PLA) is an established method for surface texturing. Defects include melt debris, unwanted allotropic phase transitions, recast layer, porosity, and cracking, leading to non-uniform mechanical properties and surface roughness in fabricated textures. An evaluation of the main laser parameters indicates that shorter pulse durations (ns—fs), fluences greater than the ablation threshold, and optimised multi-pass scanning speeds can deliver sufficient energy to create textures to the required depth and profile with minimal defects. Surface texturing improves the tribological performance of cutting tools in dry conditions, reducing coefficient of friction (COF), cutting forces, wear, machining temperature, and adhesion. It is evident that cutting conditions (feed speed, workpiece material) have a primary role in the performance of textured tools. The identified gaps in laser surface texturing and texture performance are detailed to provide future trends and research directions in the field.

## 1. Introduction

The use of hard and ultra-hard materials is in demand in the cutting tool industry because of their superior mechanical and wear properties. Tools made from these materials have a longer life and improve the quality of machined workpieces. Currently, a large area of research focuses on precision cutting, bespoke microstructural changes, surface property modifications, and texturing. These surface processes help to improve the cutting performance via friction reduction, wear reduction, reduction in planar stresses, improved chip flow, and increased tool life. Laser processing is generally considered an effective and reproducible manufacturing technique capable of surface engineering applications in hard and ultra-hard material [1,2].

This paper reviews the current state of the art methods in laser fabrication of textures on hard and ultra-hard materials which are difficult to cut due to their superior mechanical (hardness, toughness) and wear properties and the tribological performance of these surface textures. The current limitations and emerging directions of laser processing and texturing in hard and ultra-hard materials are also discussed.

To achieve a comprehensive review, the systematic review methodology based on the works of Denyer and Tranfield [3] has been utilised. This is based on five-steps: (i) formulation of research questions, (ii) locating studies, (iii) study selection and evaluation, (iv) analysis and synthesis, and (v) reporting and using results. The present review paper is aimed at students, researchers, academics, and industrialists working with lasers and hard and ultra-hard materials.

Following the systematic review methodology, the proceeding questions were formulated for the review:

(1) What laser mechanisms are required for the fabrication of surface textures on hard and ultra-hard materials?
(2) What are the main laser parameters involved in processing hard and ultra-hard materials to produce surface textures?
(3) What are the tribological improvements achievable with textured tools, the limitations, and the emerging techniques?

The first question is discussed in the introduction. Literature for this paper primarily used the ScienceDirect and Engineering Village database using “laser processing”, “laser texturing”, “texturing”, “surface textures”, “textured cutting tools”, “texture friction” as keywords, only including literature related to materials specified in the introduction. Each chapter consists of a summary table of literature to outline the study selection, followed by an analysis of the works to report the contributions to the field.

### Introduction to Hard and Ultra-Hard Materials

Ultra-hard materials are a class of materials whose hardness exceeds 40 GPa on the Vickers’ hardness scale [4]; these materials share superior properties including high thermal conductivity (greater than 800 Wm^−1^K^−1^ [5]), high wear resistance, and chemical stability. As a result, they are used in various industrial machining applications. Diamond and Boron Nitride are the hardest among materials identified in Table 1, with hardness in the range of 40–80 GPa for Polycrystalline diamonds (PCD), 59–75 GPa for Chemical vapour deposition (CVD) diamonds, and 28–44 GPa for Polycrystalline Cubic Boron Nitrides (PcBN) [6]. Hard materials are classified as materials with a hardness greater than 15 GPa, this includes Tungsten Carbide (WC), Titanium Nitride (TiN), Silicon Carbide (SiC), and Titanium Boride (TiB_2_) [7,8]. The hardness is dependent on grain size and binder composition [9].

**Figure 1:**
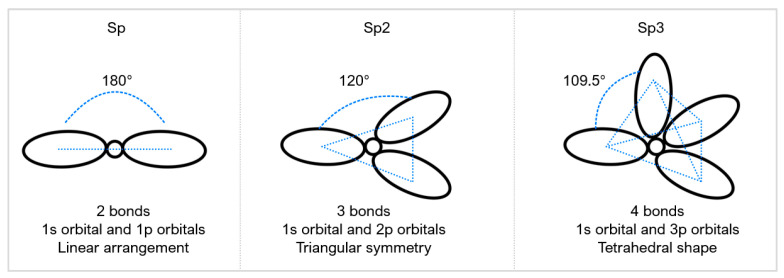
Types of hybridisations.

Diamond is an allotrope of carbon; its tetrahedral structure and strong covalent bonding are responsible for the properties many industries appreciate. For diamond formation to occur, temperatures and pressures need to exceed 1583 K and 5.2 GPa, respectively [10], which is why they form in the upper mantle, approximately 100–150 km below the Earth’s surface. Natural diamonds have a near-perfect edge roughness ideal for precision cutting; however, the natural microstructure of a single crystal can exhibit weak planes [11]. These planes arise because the tetrahedral and cubic structures have regions of fewer bonds, making the hardness directionally dependent. In PCD, the strength comes from diamond-to-diamond bonding and the hybridisation arrangement. Natural diamond mainly exhibits sp3 hybridisation where one ‘s’ type orbital and three ‘p’ type orbitals give rise to a tetrahedron shape (Figure 1). Graphite and graphitic structures have sp2 hybridisation, the notable difference being the weak interfacial layer bonding. During synthesis, it is important to know the amount, if any, of graphitisation as hardness is dependent on the sp3 and sp2 hybridisation ratio. Differences in hybridisation or phase differences lead to different material properties.

**Figure 2:**
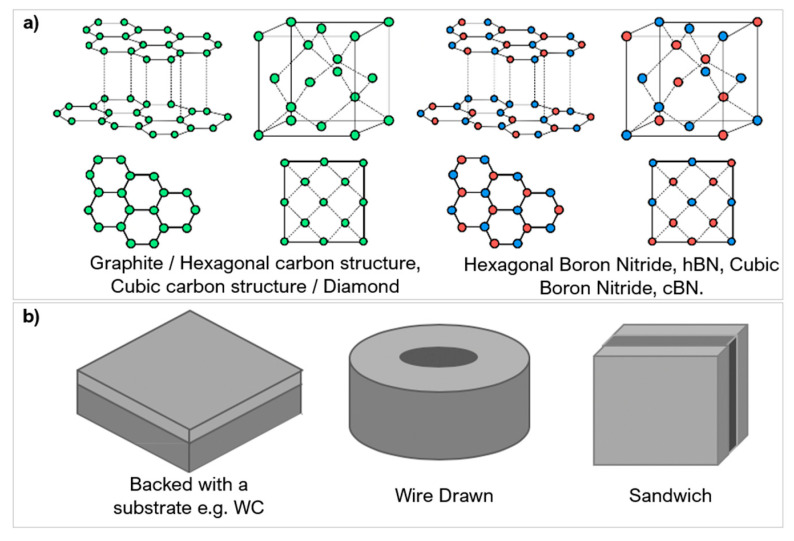
(**a**) Schematic of atomic arrangement, (**b**) possible formats for polycrystalline materials, adapted from [15].

Boron nitride (BN) rarely exists in nature with the only reported case found in micro-size quantities in Tibetan ophiolite [12] (chromium-rich rocks). It is the hardest synthetic material and is often used as a substitute to PCD in machining ferrous materials, nickel (Ni), and related alloy workpieces [5]. Amorphous BN is sp3 and hexagonal (hBN) is sp2, BN can be transitioned to have a cubic arrangement of atoms when sintered into PcBN. Figure 2a shows a schematic of PCD compared to PcBN [13].

WC, a ceramic composite, does not exist in nature. It is hard due to the covalent bonding, furthermore the binder adds toughness properties to offset the brittle nature of the ceramic. The metallic and ceramic properties are advantageous and give WC strength and durability.

The emergence of sintered particulate composites began in the 1950s for tungsten, as an alternative to diamond dies [9] and the 1970s for PCD and PcBN [14]. The composites counteract the challenges faced by natural materials and allow for flexibility in properties. Composites exhibit better wear resistance but are more susceptible to chipping when a lower binder percentage is used [5]. As the percentage increases, the toughness is improved, but overall hardness reduces. Polycrystalline structures consist of crystal grain structures randomly oriented to each other at grain boundaries. The weakest interaction occurs at these grain boundaries because of irregular bond lengths and coordination. The ability to resist deformation or damage from loads and forces is due to their polycrystalline microstructure.

**Figure 3:**
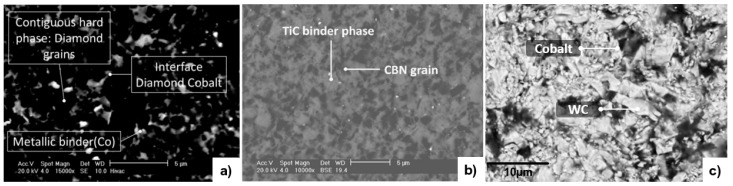
Examples of different material and binder compositions: (**a**) PCD-Co [8] (copyright permission from Elsevier), (**b**) PcBN-TiC [17] (copyright permission from Elsevier), (**c**) WC-Co.

Polycrystalline composites are sintered from fine powder grains, between 8–25 µm [16], using ceramic based or metallic binders. Cobalt is the most common binder for PCD and WC [8]. For PcBN, silicon, metallic, and ceramic compounds, e.g., aluminium (Al) or TiN are used [17]; Figure 3. The binder material acts as a catalyst to form conglomerate masses of polycrystalline structures during sintering.

High pressure and high temperature (HPHT) conditions are required, ranging from 5 to 20 GPa and 1300 to 2500 °C, respectively [4]. The stages of the sintering process can be modified to produce structures of various grain sizes, bond strengths, binder percentages, tailored for specific applications. The process of sintering on an industrial scale is expensive and energy intensive. Figure 2b shows the frequently used configurations for ultra-hard polycrystalline materials. Monitoring the sintering parameters and process is important to minimise unwanted phase transformations ([18,19]), e.g., in PCD, there is a risk of graphitic structures [20], regions of amorphous carbon [17], and nanoscopic graphite regions in the cobalt regions [21]. Sintered materials have a dense isotropic structure with no preferential orientation [8], which is favourable for hardness, wear resistance, and toughness performance. The transgranular structure causes fractures to propagate through grains and not around grains [21]. This allows for clean and precise cuts on workpiece surfaces during cutting operations.

Smaller grain sizes have higher hardness values due to the increased number of grain boundaries. Sub-micron or ultra-fine size grains (0.2–1 µm) are the hardest variant [22]. As a result, they display high hot hardness and tensile strength and can be used for positive and negative rake angle tools—with high cutting angles, allowing for better penetration of a workpiece surface [23]. The smaller grains also have better wear resistance when used in applications such as grinding as self-sharpening is easier.

Thermal processes such as laser machining can cause unwanted changes in the microstructure. Graphitisation occurs at temperatures over 900 K in PCD [21,24]. Cobalt and aluminium binders have a much lower melting temperature, the binder reaches a liquid state earlier in the matrix during the process—this liquid phase of the binder disperses in the grain structure; permanently changing it upon cooling [13,20,25]. Composite materials are also vulnerable to cracking. This is due to differences in the thermal expansion coefficients of the parent and binder material. If the values are significantly different, one material phase will expand more than the other, inducing residual stresses upon cooling (thermal mismatch). These stresses initiate micro-cracks [26], lowering the fatigue life and force resistance [27]. Table 2 provides a comparison of materials featured in the hard and ultra-hard materials and binders used.

**Figure 4:**
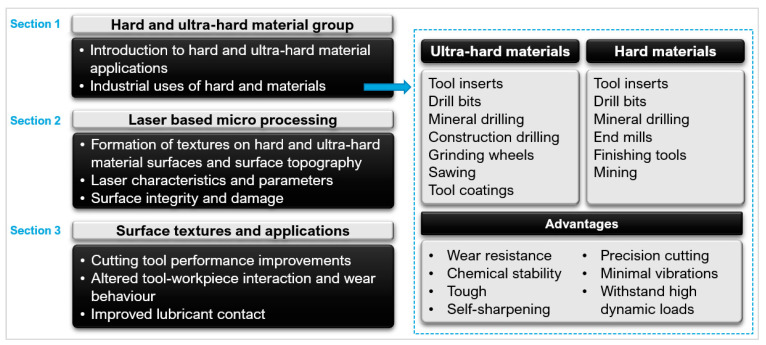
Typical industrial applications of PCD, PcBN, and WC ([21,32,34,35,36,37]).

The mechanical properties of hard and ultra-hard materials make them difficult to machine with traditional methods. The applications of these materials in the cutting tool industry include turning inserts, drills, end mills, milling, and finishing tools [28]. Figure 4 shows an overview of this and why laser micro-machining is useful in the cutting tool industry.

**Figure 5:**
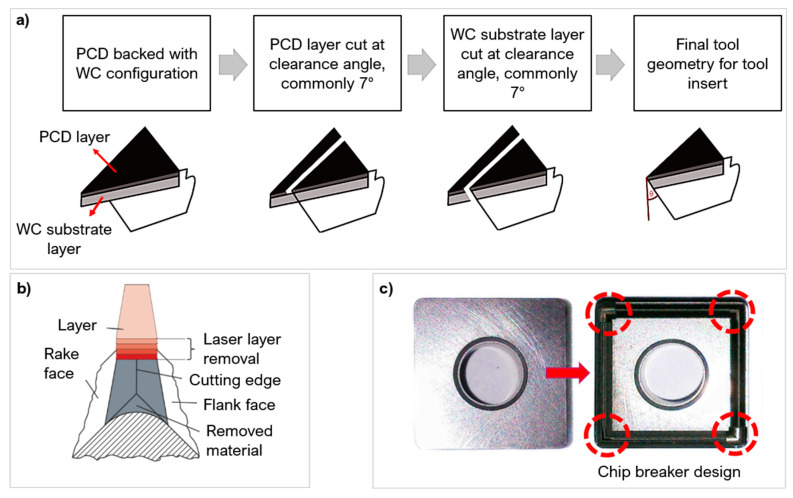
(**a**) Manufacturing stages of cutting tool insert adapted from [29] (copyright permission from Elsevier), (**b**) Cutting edge preparation by laser machining [30] (copyright permission from Elsevier), (**c**) Grooved chip breaker design by laser processing [31] (copyright permission from Springer Nature).

In producing cutting tools, manufacturers still use casting techniques by compressing fine grains into moulds at HPHT conditions, with an average processing time of 10 min per insert [29]. Processing hard and ultra-hard compacts via laser methods minimises the common drawbacks of casting such as poorer surface quality, geometric distortion, and entrapped gases [26,29]. Lasers allows for better dimensional control and the ability to create bespoke microstructural changes [13,21], as well as providing clean cuts and tailored surface roughness post HPHT sintering. Figure 5 shows examples of the use of laser ablation to manufacture cutting tool inserts to the desired shape profiles [29,30,31].

**Table 2:** Material properties of hard/ultra-hard materials. Categories labelled with a ‘/’ could not be retrieved. Aluminium has been included as a comparable reference material [16,32,33].

Properties	Diamond	Graphite	PCD	PcBN	WC	TiN	Co	Ni	Al
**Density (kgm^−3^)**	3520	2230	4100	3450	15000	5220	8900	8900	2700
**Young Modulus (GPa)**	1220	/	840	865	660	450	220	200	74
**Compressive strength (GPa)**	20	20–200	7.4	20	5	0.97	/	/	/
**Knoop hardness (GPa)**	50–100	/	/	45	11	17	/	/	2.9
**Melting temperature (K)**	Theoretical melting point as sublimation to vapour occurs at atmospheric pressures.			3246	2870	3200	1495	1455	933
**Thermal conductivity (Wm^−1^K^−1^)**	540	25–470	800	740	110	8.36	100	90.7	237
**Thermal expansion coefficient (10^−6^K^−1^)**	3.8	1.2	0.7	1.2	5.5	9.35	12.3	13.4	23.1

The application of laser processing tools is affected by the laser parameters and material’s properties (e.g., absorption, electron relaxation time, binder, and grain size). For example, smaller grain sizes are more likely to suffer from thermal mismatch causing micro-cracks throughout the processed region [38] as heat conducts more in smaller grains [39]. The binder composition also affects the results of process. Pacella et al. [40] investigated laser ablation of PCD with different cobalt binder percentages (10% and 12%). The lower binder percentage exhibited more cobalt melting and ejection without graphitisation. It also produced a better surface roughness. A key parameter in laser machining is fluence, which is the amount of energy irradiated per area on the target material [41] expressed in Equation (1). All materials have a minimum fluence level for material removal, known as the fluence or ablation threshold (further explained in Section 2.1).

(1) $$Fluence\left(J/{\mathrm{cm}}^{2}\right)=\frac{Laser\;Pulse\;Energy\;\left(J\right)}{Effective\;Laser\;Spot\;Area\;\left({\mathrm{cm}}^{2}\right)}$$

Laser micro-machining of cutting tools typically involves the creation of surface features to improve cutting performance, commonly referred to as texturing. The ultra-hard material group received less attention in texturing. Machado et al. [42] reviewed that roughly 16% of studies on textured cutting tools refers to PCD and PcBN due to scarce literature in laser ablation in these materials.

This paper aims to highlight the recent progress in laser machining of hard and ultra-hard material group. It discusses the type of laser interactions involved in fabricating textures; the effects of laser parameters on the resulting surface integrity; and the desired surface geometry [43], surface quality [44], and wear performance [45] as well as the resulting tribological changes. Table 3 lists all the relevant laser parameters and operational parameters with texturing cutting tools identified in this review.

## 2. Fabrication of Surface Textures

Laser processing provides an effective, non-contact and fast method to process and machine hard and ultra-hard materials for cutting tools [27]. The process can be described as either photo-thermal, where heating, melting, and vaporisation occurs leading to material removal, or photo-chemical, where bond brea king occurs with insufficient time for conduction to surrounding areas. Photo-thermal transformations generally take place under conditions of thermodynamic equilibrium, where heat transfer (Beer–Lambert law, Equation (2) [46]) and fluid mechanics laws govern the molten material behaviour [47].

(2) $$I\left(z\right)=I_{0}e^{-\alpha z}\;,\;\alpha =\frac{4\pi k}{\lambda }$$

where *I(z)* is the laser beam intensity at a depth of *z*, *I_0_* is the laser beam intensity at the surface, α is the absorption coefficient of the workpiece material, *λ* is the laser beam wavelength, and *k* is the material extinction coefficient.

The electron band gap of a material influences the absorption behaviour. Materials with band gaps greater than 0 eV (e.g., PCD and PcBN) require sufficient energy for electrons to be excited from valence bonds [48]. Once the energy level is reached, it causes the release of free electrons leaving electron holes behind. These free electrons further excite other bound electrons in the valence bonds. As the electrons relax, they conduct energy to the rest of the lattice causing conduction. Materials with no band gaps have free electrons, e.g., WC, the conduction process begins immediately and thus needs less energy initiate conduction.

**Figure 6:**
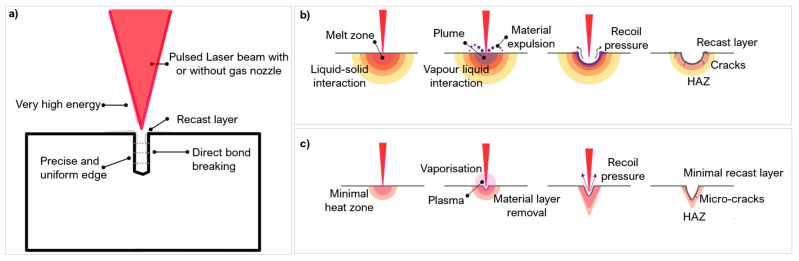
(**a**) Ablation process, (**b**) fluence ~ material ablation threshold, (**c**) fluence >> material ablation threshold.

Pulse laser ablation (PLA) is a common laser-based method used to create surface geometries on a range of materials including hard and ultra-hard composites [46,49,50,51]. PLA is a photo-thermal and photo-chemical process which uses a pulsed laser set at a fixed pulse duration. The fluence and pulse duration dictates whether the absorption is linear or non-linear. The efficiency of the process is improved with multiphoton absorption (Section 2.1). When the transferred energy reaches values above the fluence threshold, the workpiece material will ablate [44]. During this process, vaporisation, recoil pressure, and sublimation induced from the process expel material to make surface features (Figure 6).

Lasers are able to produce features and geometries in the order of 10–100 μm [52]. In special instances, nano-scale features can be made, and these are known as laser induced periodic surface structures (LIPSS) [53]. Common defects associated with PLA are melt debris, allotropic transitions, heat affected zones (HAZ), recast layer, porosity, and cracking ([54,55]) (Figure 6). HAZ is a result of excess heat conduction causing regions of the microstructure to be heat-treated, giving rise to anisotropic properties [56]. HAZ is brittle compared to the surrounding areas and cannot withstand forces. Unwanted allotropic phase transitions induce stresses, causing fragmentations of small chip grains [8]. A recast layer is the deposition of ablated vapour which leads to an uneven surface topography; it can also be caused when the recoil pressure is not high enough to expel material from the processing area [57]. These defects have an adverse effect in industrial applications and need to be minimised via parameter optimisation. Table 4 presents a summary into laser ablation parameters used to process hard and ultra-hard materials.

### 2.1. Effect of Laser Parameters on Surface Engineered Composites

The following section outlines the main laser parameters involved in PLA of hard and ultra-hard materials for the manufacture of surface geometries and textures [22], and the effect of these parameters on the surface integrity of the composites. The laser ablation process is influenced by many factors including the wavelength, laser medium, pulse duration, fluence, scanning speed, etc.

#### 2.1.1. Effect of Wavelength and Laser Type on Ablation Mechanism

**Figure 7:**
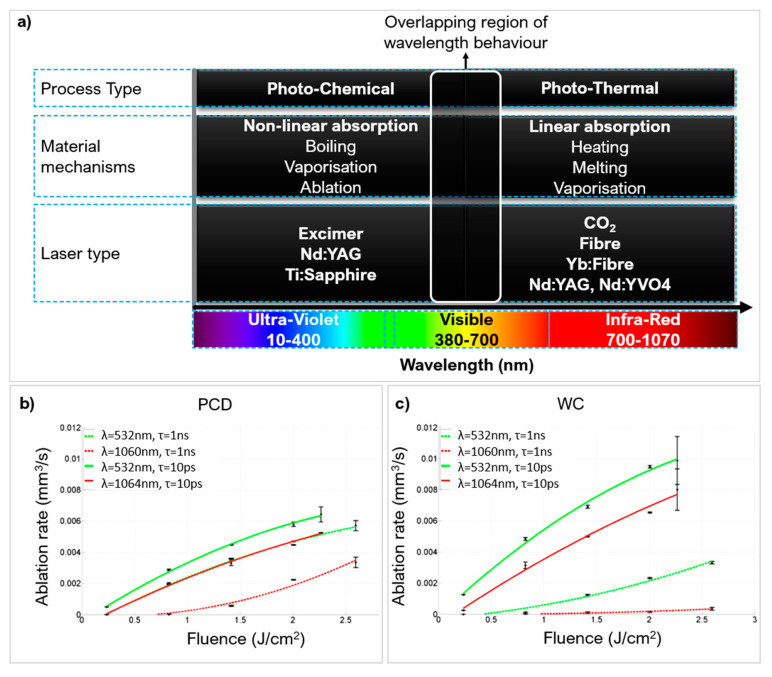
(**a**) Overview of material behaviour at different wavelengths, (**b**) Ablation rate for PCD. (**c**) Ablation rate for WC [59] (copyright permission from Elsevier).

The wavelength governs the material mechanisms and absorption behaviour (Figure 7a). A wavelength of 1064 nm is commonly used to ablate hard and ultra-hard materials because of maximised absorption [58,65]. However, the absorptivity and optical breakdown efficiency is improved with shorter wavelengths (532 nm) via multiphoton absorption even in insulating materials such as PCD and PcBN [66]. Multiphoton absorption readily initiates electron excitation and mobility. The advantages of this are higher ablation rates with better precision. Eberle et al. [59] ablated PCD-Co10% and WC-Co4.6% at 2 distinct wavelengths: 532 nm and 1064 nm. The ablation rate was consistently higher at different fluences and pulse durations when the 532 nm was used (Figure 7b,c).

**Figure 8:**
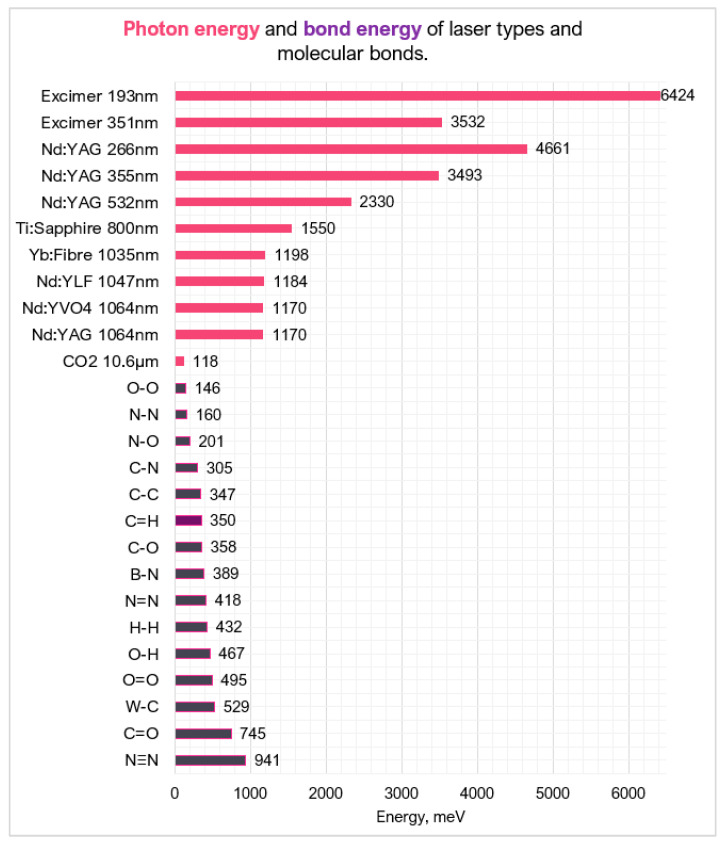
Comparative chart of laser photon energy and bond energy values [67,68,69].

The laser type/source is the medium used to generate the laser beam. The choice governs the photon energy of the beam. This is particularly important if a photo-chemical mechanism is desired as the photon energy needs to be greater than the bond energy of the workpiece material. Figure 8 compares laser medium photon energy with common molecular bonds in hard and ultra-hard materials [67,68,69].

The most widely used lasers for hard and ultra-hard materials are Nd: YAG, Excimer, and Fibre lasers. This is extended to other laser mediums such as Ti:Sapphire for hard materials (WC, TiC, and TiN), as Dumitru et al. [60] demonstrated when ablating cutting tool materials (WC-Co6%, WC-Co 10%, TiC, TiN, and industrial diamond).

Neoytterbium (Nd) lasers are near infrared (IR) lasers and can readily produce feature sizes of 20 μm [70]. There are 3 main types of Nd lasers: Nd:YAG, Nd:YLF and Nd:YVO_4_. The wavelengths vary slightly from 1064 nm to 1047 nm, respectively. Nd:YAG has a more stable refractive index. Nd:YVO_4_ can be pulsed at a high repetition rate but results in a lower energy per pulse. Zhang et al. [58] compared the effect of different Nd lasers on the surface integrity of PCD revealing that the microstructural damage is strongly dependent on the Nd laser used; for example, Nd:YVO_4_ caused cracking along the PCD substrate boundary and the Nd:YAG laser caused striations. These results were reinforced by Wang et al. [71] which also found that the higher energy of the Nd:YAG laser caused a thick recast layer on the surface due to excess energy deposition.

Excimer lasers have high pulse energy, ideal for ablation in the UV wavelength range (193–355 nm). They are an efficient medium for breaking molecular bonds; in the PCD, the HAZ can be restricted to a 1 μm region with no striations [58]. The repetition rate is limited to only a couple of kilohertz. An argon gas is required to generate the excimer laser. This gas needs to be monitored; as it ages with use, it alters the beam uniformity [70].

Since the 1980s, the use of fibre lasers has overtaken CO_2_ laser, with over 60% of laser cutting machines being fibre-based lasers [72]. Fibre lasers are reliable, with a high peak power and good optical quality [22]. They can also be modified to have different beam profiles and maintain a high power distribution [70].

Typically, frequency in the range of kilohertz (kHz) [61,73] is used to ensure the delivery of sufficient laser energy to cause ablation [60]. Fewer studies have investigated the megahertz (MHz) and gigahertz (GHz) range in ultra-hard materials. In hard materials, the MHz range can cause avalanche ionisation. Zhang et al. [74] used a 1.5 MHz frequency, UV laser, to make micro-grooves on WC disks. The plumes generated during the process rapidly expanded, ejecting vapour and material from the groove efficiently. The groove profiles were clean and consistent with small HAZ and recast layer.

#### 2.1.2. Impact of Pulse Duration on Surface Integrity

**Figure 9:**
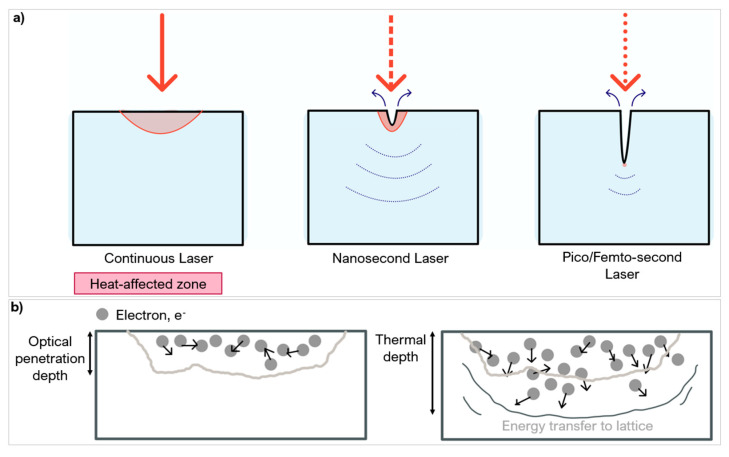
(**a**) An adapted schematic to compare the effect of pulse duration on a target surface, adapted from [70]. Shock waves are shown by the blue dotted lines. Shorter pulse durations show material expulsion, (**b**) Electron energy transfer at optical penetration depth compared to thermal depth (11mm for PCD, 5.40 mm for PcBN, 1.2 mm for WC [46]).

The pulse duration is the length of time of each irradiated pulse, it alters the amount of energy deposition and distribution onto a target material (Figure 9a) [75]. Like wavelength, it affects the absorption behaviour. Around the nanosecond (ns) regime, there is linear absorption which progressively changes to a non-linear absorption around the femtosecond (fs) regime [76].

Material response to different pulse durations is dependent on the fluence threshold, energy penetration depth and incubation factor [77,78]. Most metals and composites (including PCD and PcBN) have an electron relaxation time typically in the range of a few picoseconds (ps) [46]. If the pulse duration is shorter than the relaxation time, energy conduction is confined to the optical penetration depth and not the bulk material (thermal depth) which is beneficial in micromachining as it does not alter the surrounding microstructure (Figure 9b). Thermal damage cannot be completely avoided with shorter pulse durations even in ultra-hard materials which have a low thermal expansion coefficient. Ultrashort pulse durations (<fs regime) cause phase explosions and plasma plumes [29,62]. These plumes can exist in the processing region for several microseconds (μs) causing melting and/or reheating of the area—lowering the ablation efficiency [70].

Pulse durations similar or longer than the electron relaxation time allow for energy conduction to the material lattice structure, up to the thermal depth. This causes heating to the surrounding regions, having a direct impact on the volume of material melted [79] and increasing the risk of defects like HAZ and phase transitions [61]. Pulse durations in the range of microsecond can also lead to structural and heat related damage to the workpiece material [60] such as burning and possibly boiling [80]. However successful repeatable textures were created on PCD and PCBN arrays with no apparent thermal damage using pulse duration in the microsecond range [73] and in the nanosecond range [62], if other parameters are optimised.

Eberle et al. [44] studied the thermal characteristics after varying pulse durations in PCD. The 10 ps showed no residual graphite layer as it was ejected during the process with an almost non-existent HAZ. The longer pulse of 125 ns showed allotropes of graphitised carbon and low sp3 amorphous carbon. On the other hand, Denkena et al. [81] showed with PcBN the nanosecond pulse duration caused melting and recrystallisation of the binder phase. The femtosecond laser ablation caused less binder excavation, the formation of cubic BN (cBN), and better average surface roughness (Ra). Other hard materials (WC-Co6%, WC-Co10%, TiC, TiN) exhibited cracking and warping on surface craters in the femtosecond regime [60].

**Figure 10:**
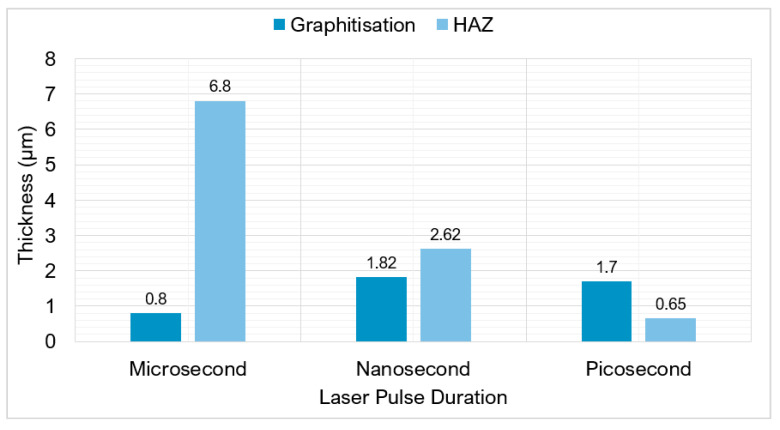
Effect of pulse duration on thermal transition in laser processing PCD (Microsecond, 100–450 μs, nanosecond, 80–125 ns, picosecond 1–10 ps, [44,50,62,82]).

Figure 10 depicts a critical comparison between the reported microstructural changes as a function of pulse duration from Eberle et al. [44], Maximilian et al. [50], Urbina et al. [82], and Okuchi et al. [62]. Using a picosecond pulse duration versus a microsecond one resulted in a 90% reduction in the HAZ thickness. In the picosecond region, there was still a HAZ suggesting that multiple factors contribute to the size of the HAZ, such as fluence, pulse energy, thermal conductivity, and gases used [58,83,84]. There was little graphitisation in the microsecond region because of the prolonged laser heat. Heat is dispersed preventing the temperature getting too high in a small region for rapid phase transitions of diamond to graphite. The use of longer pulse durations caused greater melting effects and striations. The nanosecond range appeared to be the maximum limit where large thermal changes occurred. Durations shorter than picoseconds significantly reduce the risk of thermally induced phase transformations [49]; small amounts of graphitisation can still occur because the regions are superheated during the laser interaction.

**Figure 11:**
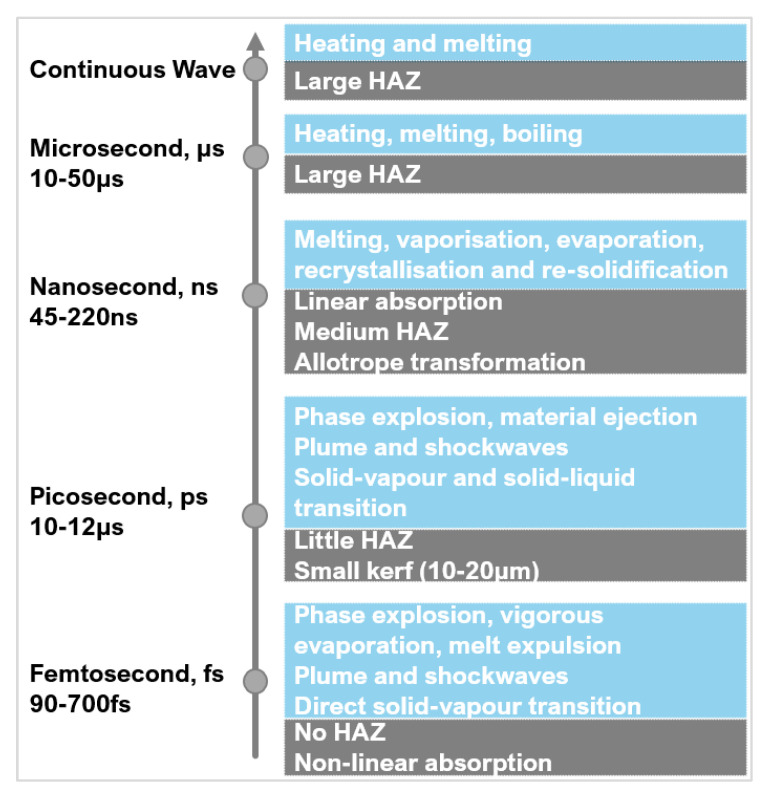
Pulse duration and processing comparison.

Studies have shown the benefits of the shorter pulse in precision ablation of hard and ultra-hard materials, as they focus energy into an extremely small region to produce surface textures. Figure 11 shows a brief overview of the processing mechanisms discussed in this section.

#### 2.1.3. Effect of Fluence on Allotropic Transition of Hard and Ultra-Hard Composites

The fluence (Equation (1)) in micro-machining needs to exceed the ablation threshold while avoiding thermal damage to the target and the surrounding regions. The fluence is increased by either increasing the pulse energy or minimising the spot size; the laser spot diameter is often in the range of 10–100 μm [71,85]. The fluence should be set to minimise the number of laser passes, as this causes multiple heating and cooling cycles in the microstructure. Repeated heating and cooling cycles are unfavourable as they increase the likelihood of internal residual stresses [27]. These stresses lead to crack formation, attributed to the thermal mismatch between hard phase and binder (e.g., WC and cobalt [58], PCD, and cobalt [86]).

**Figure 12:**
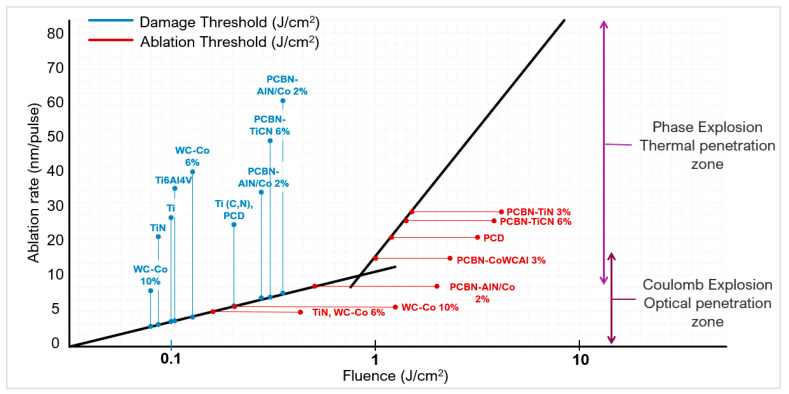
Ablative threshold of hard and ultra-hard materials [60,89,90].

The fluence threshold is dependent on the material composition (e.g., binder percentage, grain size) and the pulse duration [79]. Shorter pulse durations decrease the fluence threshold. Lickschat et al. [87], when investigating ultrashort pulse durations, found the ablation threshold of WC decreased from 0.4 J/cm^2^ to 0.26 J/cm^2^ as the pulse duration decreased from 10 ps to 0.2 ps, respectively. Fang et al. [88] using green laser light (532 nm) also demonstrated a decrease in ablation threshold on WC-CoNi from 2.5 J/cm^2^ to 0.5 J/cm^2^ when the pulse duration regime was changed from nanosecond to picosecond. Figure 12 is a comparison of the damage and ablation fluence thresholds in different materials based on a pulse duration of 150 fs, 800 nm, 1 kHz, using the investigations of Dumutri et al. [60], Zheng et al. [89], and Denkena et al. [90]. The damage threshold for PCD is lower than expected because of the binder content [44].

**Figure 13:**
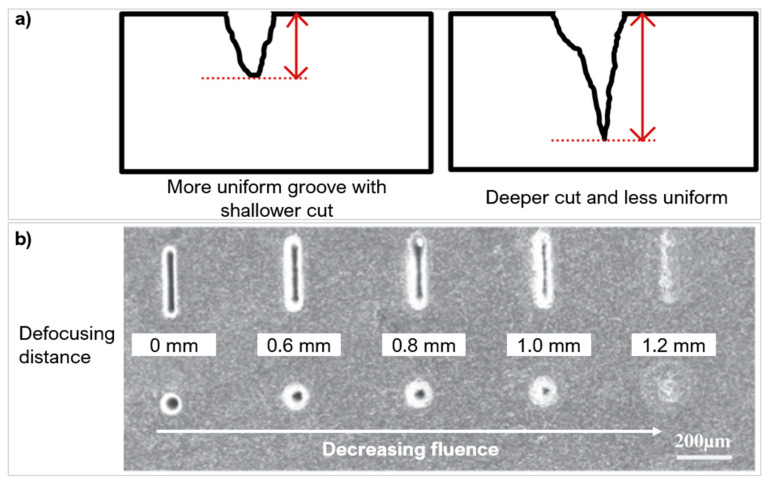
(**a**) Lower fluence compared to higher fluence, (**b**) Effect of defocusing distance (fluence) on PCD on micro-texture [92] (copyright permission from Elsevier).

To create clean surface textures, a favourable material removal/ablation rate should be reached. In ultra-hard materials, a fluence close to or less than the threshold will have a shallower material removal rate, around 1–30 nm per pulse [91]. The main phenomenon for material removal is Coulomb explosion; the high thermal energy allows ions in the lattice to repel and breakdown aiding the material removal process [76]. Surfaces are often smoother but shallower, requiring multiple laser passes. At fluences much higher than the threshold, phase explosion and plasma heating (superheated liquid) dominate, giving a material removal rate of about 100 nm per pulse [44] (or 37.5 mm^3^/min [88]). As a result, the higher fluence will cause deeper features in fewer passes, which is very efficient (Figure 13a) but often matched with an increased surface roughness. Uneven profile features and textures will alter the cutting tool behaviour and deviate from the expected or predicted performance. An optimised fluence can produce uniform micro features. Su et al. [92] showed this when creating dimples and linear grooves with a fibre laser on a PCD tool. The study changed the defocusing distance to change the fluence (alters the effective laser spot size). As the fluence decreased, this resulted in a shallower groove depth with less precise material removal (Figure 13b).

The material interaction is also more aggressive at fluences much higher than the threshold, the microstructure experiences more binder melting, binder expulsion, and crack formation [49]. The fluence also influences the depth to which the microstructure is affected. Pacella et al. [8] used a μs laser to process PcBN, at two fluences: 737 J/cm^2^ and 623 J/cm^2^. The substructure under the ablated surface remained undisturbed at the lower fluence, but the higher fluence revealed distinct boundaries of different BN allotropes in the substructure (e.g., hBN and amorphous BN). The effect of fluence on allotropic transitions is clearly shown by Denkena et al. [49] using a ns laser on PcBN-TiC with different fluences. At 3.4 J/cm^2^, there was binder melting, excavation of melt material, and binder recrystallisation on the surface of cBN. By 56 J/cm^2^; the ablation mechanism shifted towards sublimation with less melting. At 225 J/cm^2^, the surface was more homogenous with fewer regions of cBN and debris. Above 300 J/cm^2^, the surface exhibited deep cracks and strong melt debris; at 900 J/cm^2^, the surface was covered with cracks and a porous structure.

Induced phase transition of amorphous alpha BN (sp3) and hexagonal BN (sp2) can easily be generated when creating surface textures on PcBN with prolonged laser interaction or high fluence. Breidenstein et al. [93] textured PcBN with varying fluences from 3.0 J/cm^2^ to 4.4 J/cm^2^. The tools textured using 3.7–4.4 J/cm^2^ had the highest amounts of hBN than fluences less than 3.7 J/cm^2^ setting. Pacella and Brigginshaw [94] also demonstrated phase transitions when creating three different textures—linear grooves parallel to the chip flow direction, perpendicular to the chip flow direction (CFD), and a crosshatch pattern at 22.7 J/cm^2^. The crosshatch design had the highest presence of the phase transitions because of the prolonged laser interaction required to produce the texture. The hBN phase was concentrated in the deepest part of the groove.

WC is more prone to cracking defects from laser processing, unlike PCD and PcBN. The ultra-hard materials have a lower thermal expansion coefficient and higher optical penetration depth [95]. Fang et al. [57] used a ns UV laser with fluences between 10 and 20 J/cm^2^ to create textures with various geometries on WC—triangle, square, hexagon, and octagon. As the fluence increased, the amount of molten material redistribution increased in the processing region resulting in micro-cracks and porous recast layers. These effects also caused a deviation in texture morphology.

#### 2.1.4. Impact of Scanning Speed on Texture Generation

**Figure 14:**
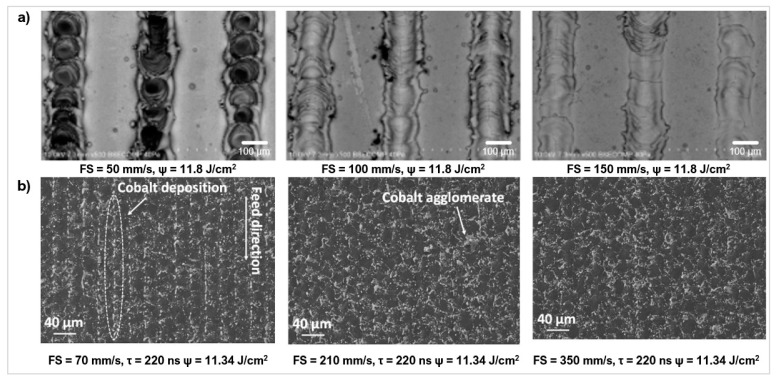
(**a**) Microgrooves on WC-Co at different processing speeds [95] (copyright permission from Trans Tech Publications), (**b**) CTM302 (PCD), resultant SEM image at different processing speeds: 70 mm/s gives Ra = 0.5 µm, 210 mm/s gives Ra = 0.41 µm [40] (copyright permission from Elsevier).

The scanning speed determines how long the workpiece is exposed to the laser beam in an area. It needs to be optimised to the material and laser beam characteristics to cause enough energy transfer for sufficient uniform material removal with limited damage to the surface. State of the art processing of PCD and PcBN materials used feed rates ranging from 2 mm/s to 900 mm/s [96,97]. Vazquez et al. [95] used a ns laser at three laser speeds (fluence of 11.8 J/cm^2^), to create micro-grooves on WC-Co (Figure 14a). The fastest speed only penetrated the very top surface layers but still transferred enough energy to cause material splattering along the texture. As the speed decreased, the depth of the groove increased with a more defined texture form.

Pacella et al. [11] investigated the effects of feed speed on the microstructure of two PCD composites with medium and fine grain sizes using a Yb:fibre laser. A speed of 70 mm/s showed a greater metastable conversion of graphite. This also caused more cobalt melting expansion, inducing compressive stresses in the surrounding regions. Experiments by Pacella et al. [98] agreed with these findings, deducing that at scanning speeds around 100 mm/s, there was a greater difference in the cobalt binder percentage distribution, a large amount of binder debris, and thermal damage [99]. The prolonged laser energy over a small region caused the binder to melt, redistribute, and redeposit. Higher speeds of 350 mm/s [40] and 500 mm/s [63] speed produced a better surface roughness because of the shorter laser exposure.

Laser scanning speed also plays a notable role in the resulting surface roughness of processed textures. An investigation by Pacella et al. [40] concluded a four-times increase in the Ra parameter in PCD materials, when the laser speed was decreased by 75%. A slower speed generally increases the thermal damage to the surface, giving rise to surface defects [71] (Figure 14b).

### 2.2. Cutting Tool Surface Textures

**Figure 15:**
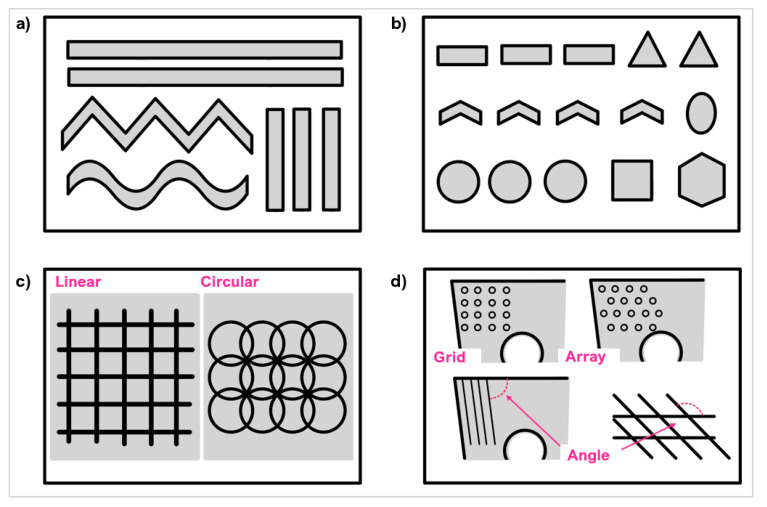
Micro-texture designs and configurations commonly used: (**a**) continuous textures, (**b**) discrete textures, (**c**) crosshatch features, (**d**) texture orientation.

Laser ablation is ideal for the micro-manufacturing of tools made from hard and ultra-hard materials for making a range of surface textures and configurations, including continuous, discontinuous, and complex geometries (Figure 15).

**Figure 16:**
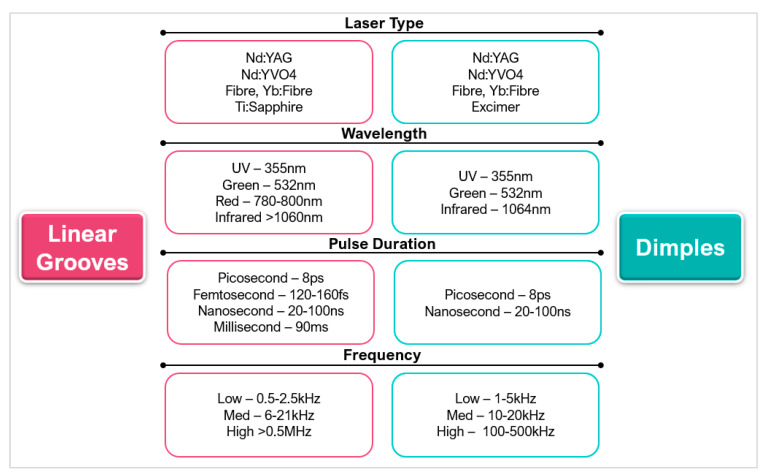
Overview comparison of laser parameter selection based on geometry type.

Laser micro-textures can be classified into three main types: linear grooves ([76,92,100]), dimples ([101,102]), and rectangular pits ([58,103,104]). Other less used textures include elliptical ([105,106,107]), sinusoidal ([108]), chevron ([109]), and hybrid/texture combinations ([110,111]). A range of micro size features can be produced with small geometrical tolerances. Adequate optimisation can also improve the repeatability and thermal damage with small HAZ in the regions of 1 μm [58]. The complexity of texture geometries when laser processing needs to be selected carefully to avoid prolonged and unnecessary laser interactions. Figure 16 gives an overview of the laser parameters used to create the micro-textures in hard and ultra-hard materials included in this review.

**Figure 17:**
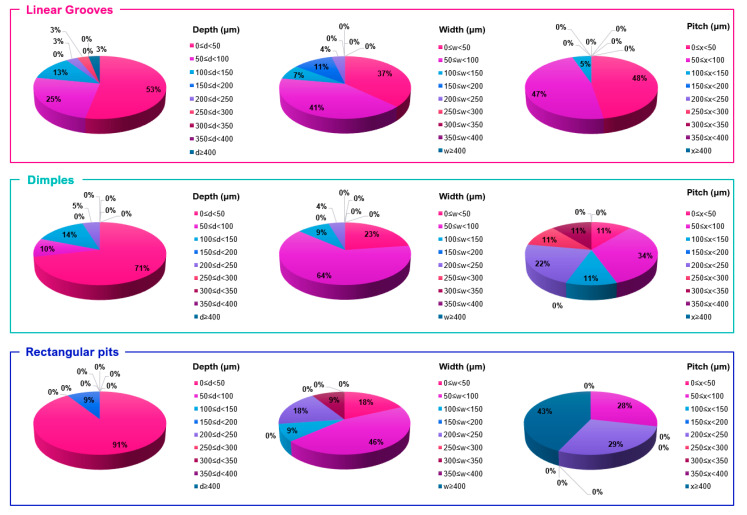
Texture dimension distribution based on the literature reviewed in this paper ([32,45,56,57,74,88,90,92,94,95,100,101,103,104,105,106,107,108,110,112,113,115,116,117,118,119,120,121,122,123,124,125,126,127,128,129,130,131,132,133,134,135,136,137,138,139,140,141,142,143,144,145,146,147,148,149]).

Figure 17 shows the distribution of texture dimensions currently used on cutting tools, separated by width, depth, and pitch. Other textures could not be included in the distribution comparison as there are fewer studies based on these textures. Comparing the dimension and size of texturing shows which geometries are frequently used and the current size limitations on texture fabrication. Across the texture types, a width of 50–100 μm and depth of 0–50 μm are most commonly used. Compared to dimples and rectangular pits, linear grooves have been made to greater depths (up to 550 μm). Whereas dimples and rectangular pits do not exceed 200 μm, the majority are made to a shallower depth, less than 50 μm. State of the art research across a range of cutting tool materials and texturing methods indicates the choice of the shallow depth is to limit thermal damage to the microstructure [112] and maintain the structural integrity of the tool [42]. The width for grooves was generally smaller compared to dimples and rectangular pits. The pitch used was also smaller for grooves allowing these textures to be denser. Dimples had the greatest variation in pitch suggesting that these textures are not restricted in terms of texture performance. The minimum size of textures was 1–2 μm depth [94] and 1–3 μm width [113]; this size was still able to produce performance improvement.

The fabrication of textures smaller than 1 μm, known as sub-micron or nano features, are just beginning to be investigated for the hard and ultra-hard material group. One type of nano-texture is LIPSS; this is formed by the plasmon interaction from polarised light [111]. The formation of LIPSS has typically been investigated on softer metals, such as aluminium alloys. However, Yunsong et al. [114] showed the possible formation of LIPSS on five different grades of carbides and concluded that the material properties (flexural strength, grain size, and phase composition) and laser properties (pulse energy, scanning speed, and scanning spacing) affected the continuity and uniformity of the structures. The main complexity in using sub-micron features resides in balancing the generation of micro-textures and nano-textures to cause a significant tribological improvement in the machining operations.

Based on the analysis of the reviewed papers, the rationale behind laser parameter selection and texture size is still based on a trial and error/statistical approach to determine the best range of settings to produce micro-textures. Statistical approaches are useful in discerning the significance of factors during processing [82,150]. The interaction between the laser and a workpiece is relatively short and complex, making it difficult to precisely predict the behaviour to achieve a desire target texture [51]. Therefore, optimisation and modelling techniques for wide scale production and manufacture are continually being developed and reviewed. Numerical modelling has shown success in predicting the outcome of laser processing. Ukar et al. [151] demonstrated numerical modelling with the use of spatial frequency analysis and differential equations to model heat transfer for surface roughness predictions, with an error between 10 and 15%. Vadali et al. [152] similarly modelled heat transfer with a fluid flow model to develop a 2D axisymmetric heat transfer model and create roughness predictions based on pulse duration and frequency, with a 12% error deviation. Advanced modelling using artificial intelligence (AI), e.g., artificial neural networks (ANN), genetic algorithms (GA) [153], and fuzzy logic (FL) [51], has recently gained attention due to the ability to collate and process a range of experimental results to find solutions to complex problem with accurate results as Stavridis et al. [154] showed in laser cladding, Biswas et al. [155] in laser micro-drilling, Phillips et al. [156] in laser additive manufacturing, and Tsai et al. [157] in laser cutting. Yousef et al. [155] predicted the pulse energy required to produce a crater of desired depth and diameter by a multi-layered artificial neural network (ANN) model. The model was able to predict the nature of material removal process with a high degree of accuracy and an average error of 2%. Numerical modelling focuses on understanding fundamental energy transfer and behavioural properties of the laser beam used and workpiece material, whereas AI does not require the integration of material science to formulate a predictive model; the methods are often referred to as black boxes because the internal modelling layers are hidden [158]. However, they are able to handle large datasets; they prove less sensitive to changes in parameters and are more accurate. These models could be used in industry to tailor the target’s materials properties by laser parameters. This would be particularly beneficial in hard and ultra-hard materials where the cost of production is extremely high.

## 3. Surface Texture Performance

The use and form of textures on cutting tools have been inspired by various biological structures [159,160] with the main aim of improving the tribological performance during machining. The presence of micro-textures on a surface reduces the contact length between the workpiece and the cutting tool causing a general reduction in machining forces, COF, and cutting temperatures to various extents in dry cutting. Numerous studies have supported these improvements across a range of cutting tool materials: PCD (Ghosh and Pacella [100]), Su et al. [119]), WC (Wang et al. [161]), Titanium alloys (Niketh and Samuel [127], Costil et al. [147]), PcBN (Pacella and Brigginshaw [94]), aluminium alloys (Liu et al. [121], Mishra et al. [109]) and Inconel 718 (Pang and Wang [124]). Textures also act as reservoirs for lubricant and debris.

**Figure 18:**
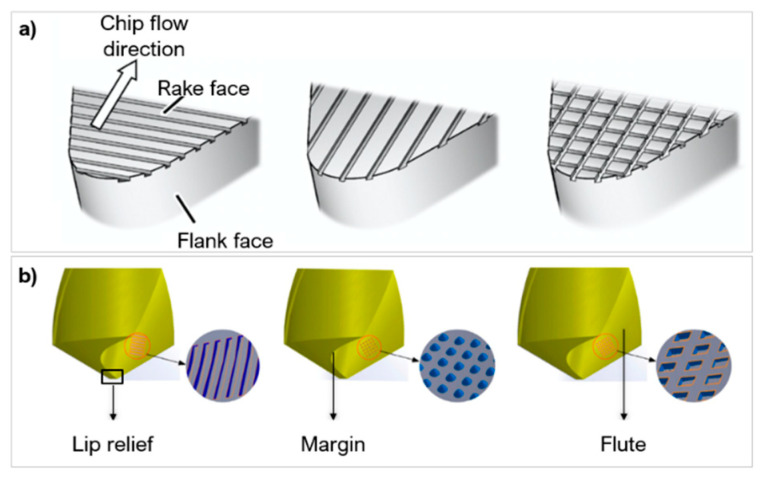
(**a**) Microgrooves generated on the rake face on turning cutting tool; arrow indicates chip flow direction [162] (copyright permission from Elsevier), (**b**) Micro-textures on rake face of drill bit (below) [124] (copyright permission from Elsevier).

Different locations on a tool can be laser textured: the rake face, flank face on single point cutting tools or the margin, flute, lip relief on multiple point cutting tools (Figure 18 [124,162]). On single point cutting tools, the rake face is typically textured as it is the initial region of chip-tool interaction; changes to the contact length and form have a greater effect on friction, wear, adhesion, and lubrication [112,118]. Flank face texturing improves the overall wear resistance; textures in this region help to remove debris and prevent inclusion scrapping between the tool and chips [121]. Sugihara and Enomoto [122] showed a reduction in flank wear from 170 μm to 120 μm, on a WC face milling tool with the use of micro-spikes. In drilling tools, the margin is often textured, as it is in sliding contact with hole surfaces and chips flow over this region. Textures cause less adhesion and improve shedding of adhered chips [104]. The flute is textured less because it is less suspectable to adhesion, but texturing the surface aids in lubrication, enabling chip removal from the drill tip [163]. Niketh and Samuel [127] demonstrated a reduction in torque forces by 10% and 12% when the flute and margin where textured, respectively. There was also less burr formation on the holes drilled when both the flute and margin was textured. The lip relief develops the most wear and heat accumulation during drilling. However, the area is small, texturing will affect the mechanical strength and integrity of the drill tip [104]. Table 5 presents a summary of research contributions of laser texturing in hard and ultra-hard materials and their effect on tool performance.

### 3.1. Effect of Textures on Friction Properties

Micro-textures on a cutting tool insert reduces the contact length between the workpiece and tool. Current state of the art research has shown the effectiveness of textures in reducing the COF and cutting forces as the surface structures allow for better heat dissipation [94] and storage of wear debris [120] and provide space for lubrication [126]. Textures also limit fluctuations, causing a reduction in chatter marks [166]. The friction force generated in cutting tools can be measured using a dynamometer and characterised using Equation (3) [130]. This formula is used to determine the COF at the tool-chip interface, and it is applicable for single point turning operations.

(3) $$\mu =\tan\left(\eta \right)=\tan\left(tan^{-1}\left(\frac{F_{y}}{F_{z}}\right)+\gamma \right)$$

where *μ* is the COF, *η* is the friction angle, *γ* is the rake angle, *F_y_* is the thrust or feed force, and *F_z_* is the tangential or cutting force.

**Figure 19:**
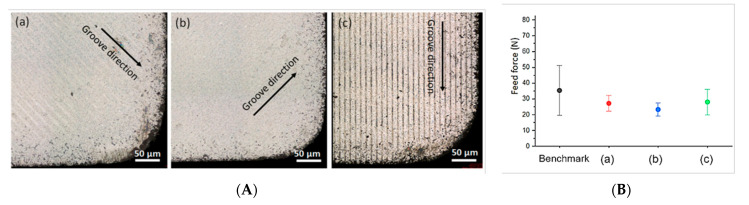
(**A**) Linear grooves on rake face, (**B**) Feed force of micro-textured tools compared to untextured tool after a sliding distance of 2.785 km [100] (copyright permission from Springer Nature).

Texture orientation and geometry strongly affect the frictional behaviour. Ghosh and Pacella [100] created linear grooves (width = 7 μm, depth = 260 nm and spacing = 20 μm) on the rake face of a PCD tool using a nanosecond fibre laser to investigate the effect of texture orientation in turning Al 6082. Three orientations were investigated: parallel to CFD, perpendicular to CFD and parallel to the main cutting edge (MCE) as shown in Figure 19A. The micro-grooves reduced the cutting forces (Figure 19B) and improved the frictional behaviour of the tool, with adhesion reduced by 59.36%. The parallel grooves reduced the COF by 14.28%. The force was reduced by 23% and 11.76% with the perpendicular and parallel groove direction, respectively. The perpendicular groove direction also improved the stability of the feed force.

**Figure 20:**
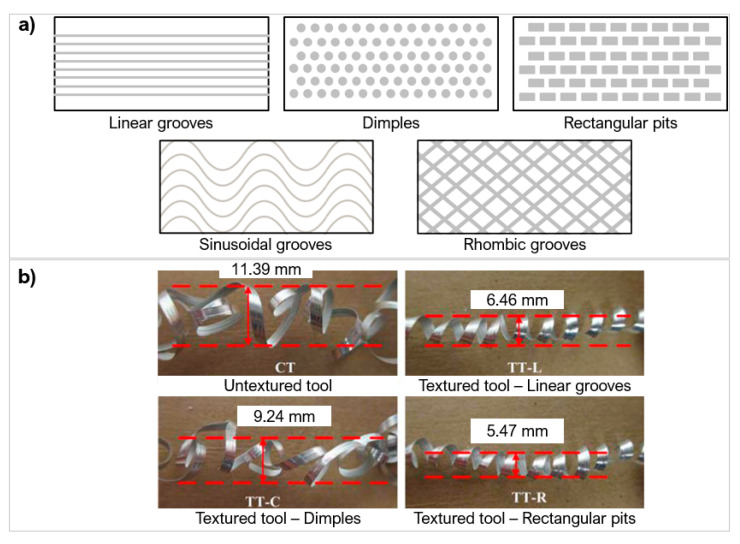
(**a**) Types of micro-textures: linear grooves, dimples, rectangular pits, sinusoidal grooves, rhombic grooves, (**b**) Chip formation from the different texture geometries (right) [103] (copyright permission from the American Society of Mechanical Engineers).

The geometry of textures alters the contact length between the tool and workpiece and the ability to hold wear debris. Xing et al. [103] used a Nd:YVO_4_ laser to create three different geometries on the rake face of a WC tool: linear grooves, dimples, and rectangular pits, at a depth of 10 µm with a 20% areal density (Figure 20a). The tool was used to turn Al_2_O_3_. One of the biggest effects of changing the micro-geometry was on the chip formation structure. The rectangular pits created the smallest and the most uniform chips out of the 4 conditions (Figure 20b). The dimple texture produced jagged, uneven chips which are unfavourable but were still smaller than the untextured tool. The linear grooves and rectangular pits created very uniform chip diameters, almost half the diameter of the untextured tool. The rectangular pits generated the smallest cutting force and best lubricity of chips. Pacella [31] also demonstrated altered chip formation in WC using a chip breaker design manufacturing with a nanosecond laser (Figure 5c). The chip breaker design resulted in two main chip formation mechanisms when machining AISI 1040. A lamella chip was formed due to excess shear strain causing cleavage cracks. A novel chip shape identified as brush-stroke type was also formed from the localised temperature increase and the tool chip interface causing thermal softening allowing for large plastic deformation.

The differences in cutting performance from texture geometries were also confirmed by Zhang et al. [108]. The study created linear, sinusoidal, and rhombic grooves on WC (YG8) of width 159.6 μm and depth 14.6 μm (Figure 20a) to machine Ti_6_Al_4_V via nanosecond laser. The textures stored debris, dissipated heat effectively, and decreased the occurrence of bond wear. The linear and wavy groove reduced the COF by 34% with the least amount of adhesion, both reducing the amount of surface bonding of the titanium alloy and the furrow effect. FEA analysis in this study showed that the sinusoidal texture produced the highest surface temperature distribution, and the rhombic texture showed the greatest amount of stress among the textures.

The width to depth ratio (area ratio) of the geometries also influences the frictional properties. Generally, a higher width-to-depth ratio decreases the COF to a greater extent [74]. However, there has not been a thorough analysis to fully understand why this is the case.

**Figure 21:**
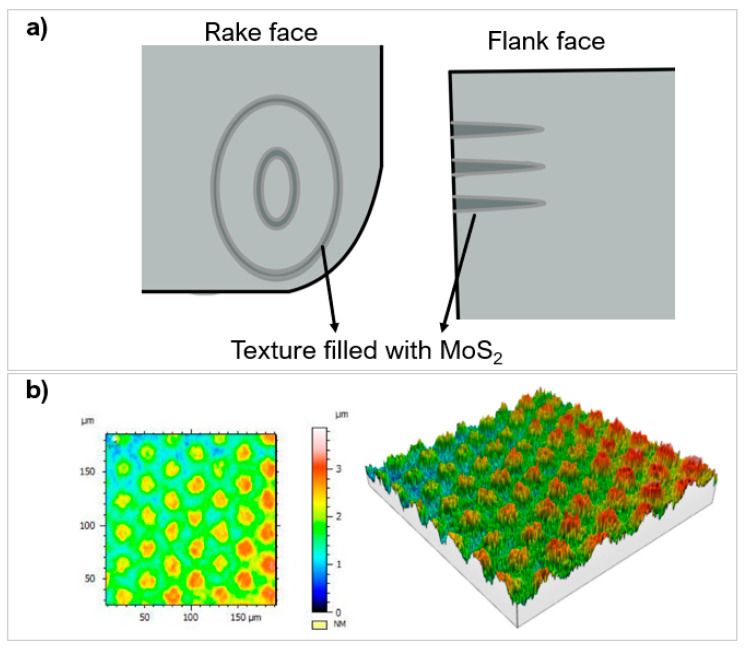
(**a**) Schematic of elliptical grove textures on rake and flank face, filled with MoS2 adapted from [107], (**b**) Honey-comb geometry created on a H10S WC tool [135] (copyright permission from MDPI AG).

There have been fewer studies with unconventional geometry profiles ([109,167]) and texture combinations ([111,168]); these provide their own advantages compared to the standard geometries [118]. Ze et al. [107] used a femtosecond laser to create elliptical and linear grooves filled with molybdenum disulphide (MoS_2_) on WC lubricant for turning hardened steels (Figure 21a). The elliptical geometry was chosen because crater wear typically exhibits this shape. The textures were placed 150 μm from the MCE in an attempt to reduce the general wear of the tool. The elliptical shape outperformed the linear grooves reducing the forces by 20%. The shape allowed for the lubricant to be released easily and smear on the rake face, reducing the contact length from 1 mm to 0.8 mm at the tool interface compared to the linear design. Orra et al. [106] also showed incremental improvements with elliptical grooves over linear grooves on a Al_2_O_3_ tool coated with TiC. The textures improved the cutting force, COF, and cutting temperature and minimised the COF by 11.9%. A honey-comb like texture was investigated by Pakula et al. [135], on WC and sialon ceramics using a picosecond Nd:YVO_4_ laser at two wavelengths. The tribological performance was tested on a pin-on-disc tester with an Al_2_O_3_ workpiece material. The textures had a width of 30 µm and a depth of 650 µm (Figure 21b). The tribological performance was similar to standard textures; however, this texture configuration reduced the variability of COF by 20%.

**Figure 22:**
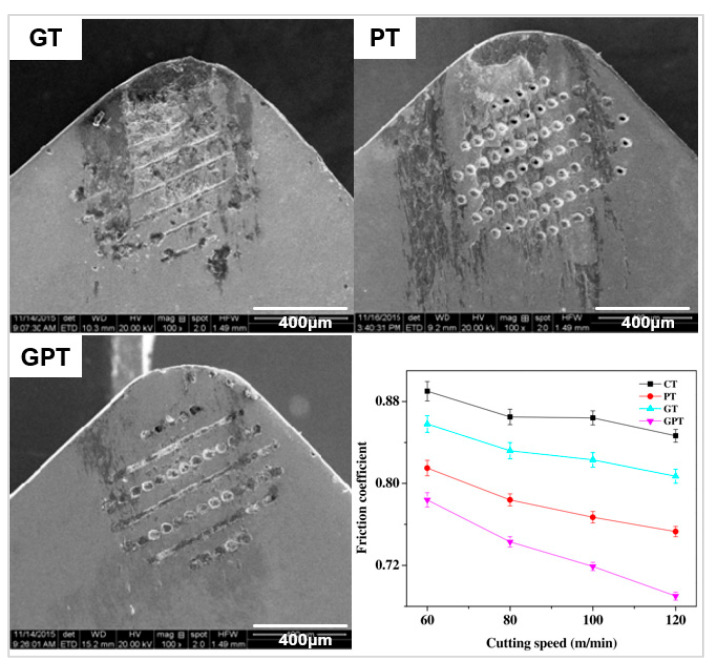
Wear track on WC rake face at 120 m/min after 150 s cutting with different texture geometries: GT-Grooves, PT-Dimples, GPT-Hybrid texture. Comparison of COF at tool-chip interface [110] (copyright permission from Springer Nature).

Hybrid textures use two or more micro-textures on a tool. Sun et al. [110] presented a novel investigation by manufacturing hybrid textures using an Nd:YAG laser on WC tools to turn 1045 steel. The cutting performance was compared to the benchmark and non-hybrid textures. The dimples, grooves, and combination design had a width of 40 µm, depth of 50 µm, spacing of 100 µm and were placed at a distance of 150 µm from the cutting edge. All the textures improved the cutting forces, COF, cutting temperature, and surface roughness of the machined parts. The hybrid texture outperformed the other textures at higher feed speeds (greater than 100 m/min) showing the potential in the application of laser texturing to improve the machining performance by increased process efficiency. Using the hybrid textures reduced forces and temperature by a greater amount when compared to the untextured tool and the benchmark tools of dimples and grooves, Table 6 shows a comparison of the reduction in forces and temperature. The hybrid texture also reduced the wear track and adhesion by a greater amount compared to the individual textures as shown in Figure 22. The improved performance from the hybrid texture was attributed to chips moving over the different textures on the tool, and this varied the contact length and pressure distribution.

The critical analysis of the reported research showed that different texture geometries result in different performance outputs. The texture geometry should be selected to cover enough of an area to maximise the reduction of COF and adhesion during the turning process. The improvement in tribological performance is not solely affected by the texture geometry but also by the texture size and area density [169].

The area density is the percentage of the tool that is covered in micro-textures. The density of textures has a greater effect on the variability of the COF than the reduction of the COF. Wu et al. [45] investigated the effect of surface texturing on the friction with the Taguchi method using speed, load, and area density as functions. Both dimples and linear grooves were created on a WC tool with diameter/width of 50 μm and depth of 100 μm. Three area densities were used by changing the spacing of the texture: 25% (200 μm), 20% (250 μm), and 16.7% (300 μm). The grooves and dimples both reduced the COF by 30–35% and 15–20%, respectively. The higher area density improved the stability of the COF by 90%. Wakuda et al. [128] and Hua et al. [149] also came to a similar conclusion with the study of friction reduction in textured tools using dimples. The density of micro-dimples with diameters of 40, 80, and 120 μm was changed between 5–30%. The maximum reduction of COF was from 0.12 to 0.10, and the best anti-friction effect was found at a diameter of 100 μm and at densities of 12.5% [128] and 19.6% [149]. Another conclusion of the studies found that dimples with the same areal densities but larger diameters decreased the COF in sliding contact applications, as this changes the contact load between the pin and texture based on the Hertzian stress theory [148].

**Figure 23:**
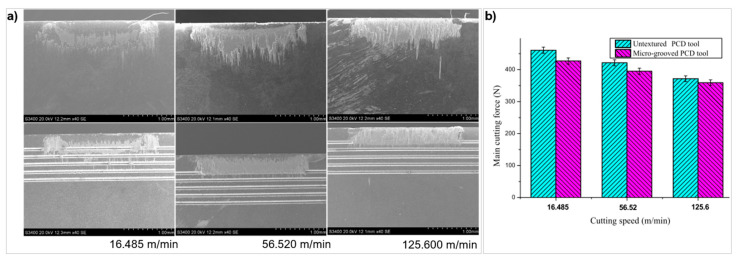
(**a**) Wear of PCD tool—Top flank face and bottom rake face after 2.758 km, (**b**) Comparison of cutting forces with textured and untextured tool [119] (copyright permission from Elsevier).

The frictional performance is also affected by the feed speed of the cutting operation. At lower speeds, typically less than 100 m/min [103], forces are higher due to deformation from strain hardening. As the feed speed increases, the temperature increases, lowering the shear strength of the workpiece known as thermal softening. This effect causes chips generated to flow easily over the rake face. This also means the extent of COF reduction is lower at higher velocities. This can cause chips to squeeze and adhere to the textures and can cause high contact stresses as chips hardened, as Xing et al. [103] showed with PCD when turning aluminium alloys. These newly formed chip regions also act as a secondary cutting edge; if these regions are sharp, this results in an increase in friction. Kawasegi et al. [113] found that speed was a factor in the reduction of the COF even with micro-textures when turning Al 5052. Linear grooves, 2.2 μm wide and 150 nm deep, were created parallel and perpendicular to the CFD. Both texture directions reduced the COF but only at speeds greater than 300 m/min. At speeds lower than 300 m/min, there was a large amount of adhesion effectively burying the textures, which made the tools act similar an untextured tool. Su et al. [119] also confirmed the dependence of feed speed on texture behaviour with linear grooves on PCD tool to turn Ti_6_Al_4_V. A fibre laser made grooves with width 56.5–60.4 μm, depth 51.5–55.4 μm, and spacing 83.9–86.4 μm on the rake face. The textured tools had a consistently better tribological performance across speeds between 16.5–125.6 m/min; there was a greater reduction in the main cutting force at the lower speed of 7.6% compared to the faster speed where the reduction was 4.7% (Figure 23). The wear of the micro-grooved texture gradually built-up. A TiC protective layer was also formed due to the reaction of carbon in the PCD and titanium in the workpiece.

Overall, the two of the main factors that affect the performance of friction are the texture dimension and the density of the texture on the tool. A comparison of geometries and their performance shows that rectangular pits and elliptical grooves perform the best at reducing cutting forces in turning by 35%. In turning operations, dimples stabilise BUE, whereas grooves tend to destabilise BUE. The comparison of more geometries using hybrid combinations and non-standard cross-sections on tools to control BUE, chip formation, and force stability still need further investigation, particularly for ultra-hard composites. This includes studying the ratio between the depth and width of textures and how they alter the effectiveness in improving the tribological performance. The effect of the texture location on the tool and the extent of workpiece interaction to texture geometries has also not been explored in detail. Furthermore, there is a lack of research in the identification on optimal texture geometry in machining due to the fact that the required performance is strongly affected by the workpiece material and the specific machining application. Future work in micro-texturing is moving towards modelling to effectively functionalise the textures and accurately predict wear rate, COF, and adhesion.

### 3.2. Effect of Textures on Wear Performance

**Figure 24:**
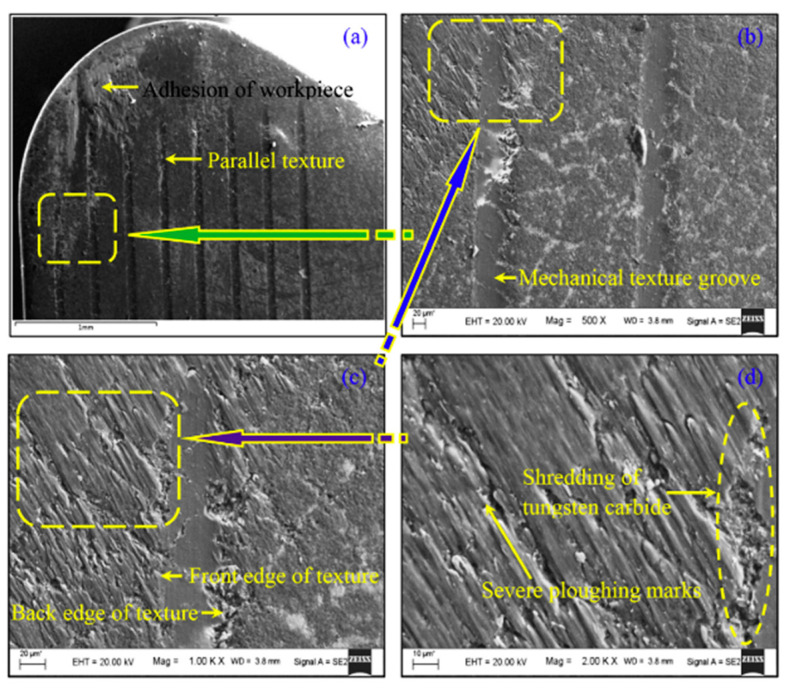
(**a**) Rake face of parallel micro-textured cutting tool on WC after 900 s of machining H-13 steel workpiece, (**b**) individual mechanical micro-texture groove, (**c**) front and back edge of texture groove, (**d**) severe ploughing marks [141] (copyright permission from Elsevier).

The main defects of wear in textured tools are material shredding, ploughing marks, and texture damage [141] (Figure 24). However, micro-textures still provide significant reduction in crater and surface wear, as Gajrani and Sankar [162] reviewed in various cutting operations including turning, drilling, and milling [122]. Textures act as micro-traps; these capture debris preventing the formation of a secondary cutting edge during the process. Therefore, the area and texture geometry will affect the ability to capture wear debris. Liu et al. [121] demonstrated this with the creation of linear grooves on WC tool with a femtosecond laser to turn Al_2_O_3_ workpieces—groove (width = 50–100 μm, spacing = 50–100 μm, and distance from the MCE = 75–100 μm). The textures improved the wear resistance of the tool and resulted in a better flank wear resistance. The configuration with grooves closest to the MCE and 75 μm groove width provided the best wear performance on both flank and rake faces. Zhang et al. [170] also studied the influence of micro-textures on wear mechanisms on WC-Ti/Co. Micro-dimples and micro-grooves with a depth of 3–10 μm and width of 80–200 μm were made on the rake face to turn AISI 1045 steel. The textured tools had better tribological properties, but the dimples performed slightly better under faster cutting speeds (200 m/min). The improved interaction at the tool-chip interface reduced the diffusion wear, as there was less diffusion of iron to the workpiece which was characterised by Energy-dispersive X-ray spectroscopy (EDX) analysis.

**Figure 25:**
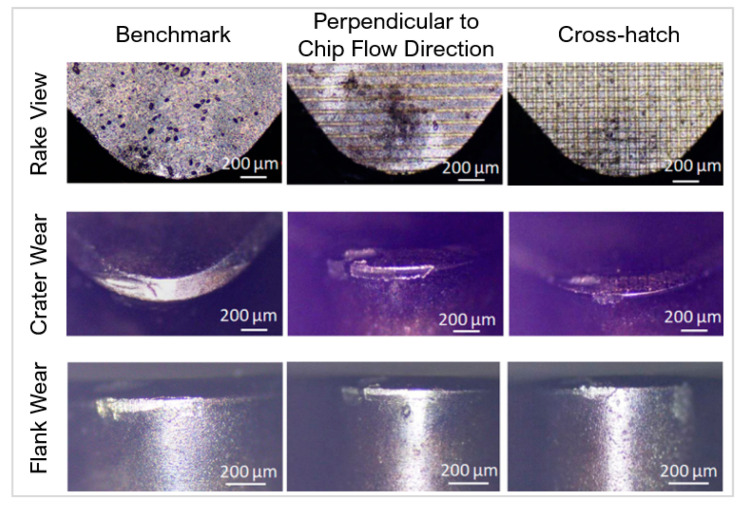
Wear mechanism on PcBN textured tools [94] (copyright permission from Elsevier).

The reduction in wear has also been shown in PcBN materials. Pacella and Brigginshaw [94] used micro-textures to enhance the wear performance of PcBN turning tools on AISI 51,200 with a depth of cut of 0.2 mm. Three linear groove patterns were manufactured using a nanosecond laser: parallel to CFD, perpendicular to CFD, and a crosshatch pattern (width = 30 μm, spacing = 75 μm and depth = 1–2 μm). The laser process produced hBN which acts as a solid lubricant aiding the heat dissipation process in the area of cut. Texturing the chamfer changed the chip behaviour in the deformation zone. The parallel grooves decreased the wear performance. The perpendicular and crosshatch both showed better wear resistance on the flank face with difference mechanisms (Figure 25). The perpendicular grooves showed purely crater wear on the chamfer edge whereas the crosshatch presented wider but shallower crater wear with localised chipping on the flank face. This is likely due to the raised stress concentrations from the texture design.

**Figure 26:**
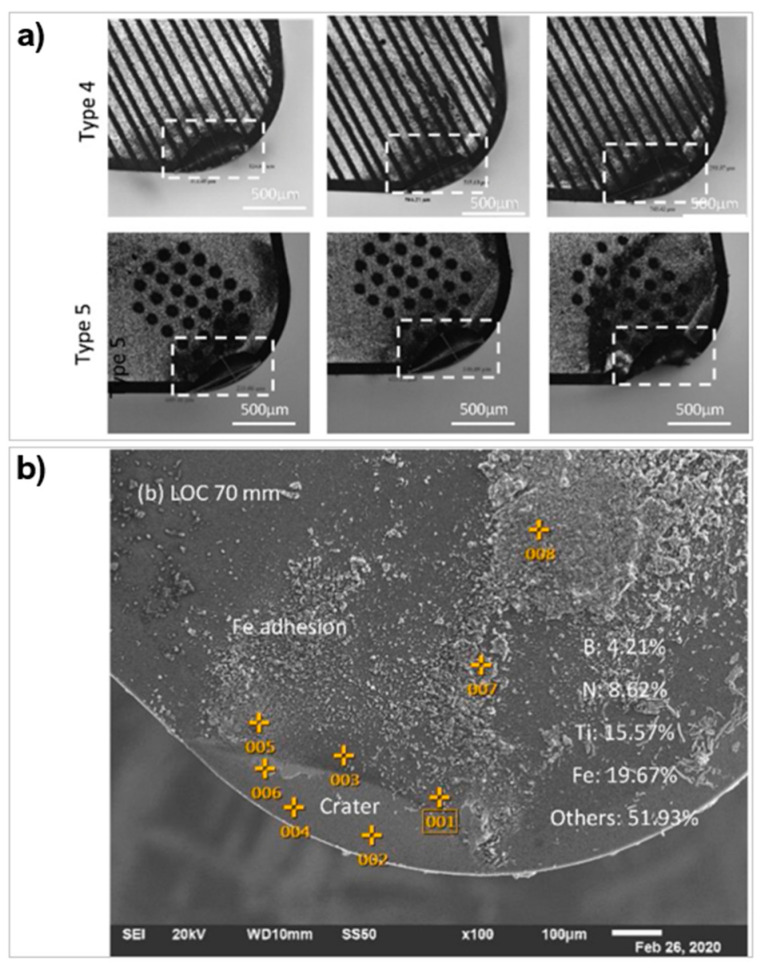
(**a**) Example of crater wear progression on two of the textures created. Type 4: width 35 μm, spacing 70 μm, depth 10 μm. Type 5: width 60 μm, spacing 150 μm, depth 15 μm, (**b**) Chip adhesion and crater wear on the rake face of PcBN tool [164] (copyright permission from Elsevier).

The improvement in wear resistance was not always evident. Law et al. [164] created 12 configurations of micro textures on the rake and flank face on PcBN to turn bearing steel. The micro-textures, fabricated using a microsecond fibre laser, were parallel grooves, perpendicular grooves, and dimples (width = 35–60 μm, depth = 10–25 μm and spacing = 70–150 μm). Crater wear was present on all the textures. Textures near crater wear did not improve the resistance to further deformation (Figure 26a). The study also found that the chip formation and surface finish of the workpiece was independent of the texture type. The contrary performance was accredited to workpiece material used and the low cBN content (45 wt%) of the tool.

The binder composition of the cutting tool affects the wear performance. Liang et al. [56] compared the wear behaviour of WC-Co and WC-Ni_3_Al. Linear grooves of depth 100–110 μm and spacing of 150 μm were made with a nanosecond laser. A reciprocating sliding test with a Ti_6_Al_4_V sphere was used to study the mechanical performance; contact loads varied between 50–200 N. Both materials suffered wear from an initial breakage of the micro-groove edge and propagating to the region between the grooves. The WC-Co tool then exhibited diffusion of the cobalt binder and WC grain growth near the micro-groove, subsequently followed by severe breakage of the groove edge. The WC-Ni_3_Al tool showed better wear resistance with less binder diffusion, less grain growth of WC and slight breakage of the micro-groove edge. Fang et al. [88] found a similar result when comparing the wear performance of WC-Co and WC-CoNi tool. There was a similar initial wear point, but the WC-Co tool showed more binder breakdown from abrasive wear of the workpiece debris.

The wear mechanism is dependent on texture dimension and tool material. This is evidenced by the changes in the type of occurring wear, for example, crater wear and shedding is more likely at shallower depths, whereas smaller texture densities are less likely to cause abrasive wear mechanisms [132]. The workpiece material and binder are also influential factors in wear; harder binder compositions exhibit less binder breakdown from abrasive wear and less binder diffusion to the workpiece.

### 3.3. Effect of Textures on Adhesion Properties

Adhesion of workpiece to the tool is a real issue for cutting tools in operation as it reduces the tool life and overall performance. During material removal operations, chips produced often adhere to the cutting tools. This changes the tool chip interaction and creates an uncontrolled BUE. This phenomenon occurs when the cutting temperature reaches the material softening point or a high enough force is applied for example with a negative rake angle. This thermal softening causes chips to be ductile and flow over the tool easier. As the chips harden, they form sharp regions and a secondary cutting edge on textures leading to a poorer surface quality on the workpiece [121]. Adhesion is heavily dependent on the workpiece material, but texturing can reduce its extent because of the reduced contact length [100]. Aluminium-based workpieces quickly reach temperatures that cause ductile behaviour causing chips to adhere to a tool [113], whereas Ti_6_Al_4_V workpieces are more likely to chip away with the pieces chemically reacting to the tool material [42]. The existence of micro-textures reduces the occurrence of surface bonding of titanium alloys and reduces the furrow effect [118]. The adhesion of titanium chips also occurs in the form of a reaction with carbon in PCD and titanium on a Ti_6_Al_4_V workpiece. The reaction forms a TiC protective layer [119]. TiC has high wear resistance and low friction; in some cases, this could be beneficial in improving the surface quality of the workpiece.

**Figure 27:**
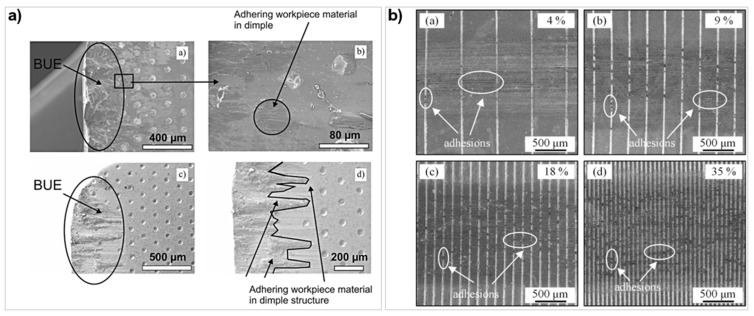
(**a**) Crater wear on cutting tool and BUE adhesion on the dimple texture after a cutting length of 4030 m [125] (copyright permission from Elsevier), (**b**) Adhesion wear on WC textured surface with W-S-C coating [120] (copyright permission from Elsevier).

Xing et al. [103] fabricated three texture types on WC via laser processing: linear grooves, dimples, and rectangular pits, and they used them to turn Al 6061 workpieces. At speeds above 219.6 m/min, the workpiece softened causing chips to easily flow over the rake face. Chips squeezed into the textures, which increased the contact stress with some chips hardening onto the edges of the micro-textures. The rectangular texture geometry had the best lubricity and prevented chips from being stuck in the texture edges. There was less adhesion on the textured tool, and the structure geometry was still clearly visible compared to the circular geometries. However, Kummel et al. [125] found that circular geometries performed better than grooves, in terms of adhesion. The micro-texture created in the study were dimples (diameter = 50 μm, depth = 20 μm) and micro-grooves (width = 50 μm, depth = 20 μm) on WC (K10) tool with nanosecond laser to turn 1045 plain carbon steel. The feed speed of the turning tool was varied between 50–150 m/min. The dimple texture showed the smallest adhesion particularly on the corner radius (Figure 27a). At lower velocities, there was visible BUE that was unstable and quickly deteriorated from the surface. The micro-groove texture caused a repeated formation of chip adhesion and chip breakage; this cycle resulted in more wear on the cutting tool. However, the dimple texture helped to stabilise the chip flow behaviour as there was a better mechanical interlock between the chip and micro-texture. This interlocking was only at the edges as focused ion beam (FIB) characterisation showed little adhesion at the bottom of the dimples.

The area density of textures also affects the adhesion behaviour of the chips. Meng et al. [120] used a nanosecond laser to manufacture linear grooves with different area densities: 4%, 9%, 18%, and 35% on WC to perform sliding tests on AISI 316 steel. The adhesion was in the form of particulate agglomerates at the micro-grooves (Figure 27b). As the texture density increased, there was more adhesion between the grooves and in the pitch spacing; this was also paired with an increase in COF, and this was linked to more textures in close proximity causing derivative cutting [171]. Law et al. [164], on the contrary, demonstrated that despite various textures on a PcBN cutting tool, the amount of adhesion was similar to an untextured tool when machining bearing steel. The tool had a high adhesion of iron characterised by the low boron and nitrogen content in those regions (Figure 26b). When comparing rake face and flank face textures, there was less adhesion on the flank face. The general results of the study were not to expectations possibly due to the workpiece material containing higher amounts of iron compared to aluminium and titanium alloys.

Adhesion wear has also been investigated with multiple point textured cutting tools (e.g., in drilling). Pang and Wang [124] textured Inconel 718 drill bits with linear, dimples, and rectangular holes (Figure 18b). The reduction in processing temperature and drilling forces was not as severe in single point cutting but a better tool life was still shown [104,127]. There was no large ploughing near the MCE. The adhesive build-up on the drill bits was similarly influenced by the cutting speed. At low speeds, chip adhesion was thicker. These layers are unstable and quickly chipped onto the workpiece. Drill bits textured with dimples reduced the amount of this adhesion because of the lower sliding friction. Ling et al. [104] textured the drill bit margin with rectangular pits with 10% and 20% density to drill 9.5 mm thick Ti_6_Al_4_V. Both densities showed significantly less adhesion contributing to the longer life of the tool of 35.9% and 22.0%, respectively, which in this case was measured with the number of holes drilled.

Niketh and Samuel [127] showed similar improvements when texturing the flute and margin of WC drill bits on Ti_6_Al_4_V. Dimples of diameter 90 μm, depth 40 μm, and spacing 80 μm were formed with a nanosecond laser. The dimples were effective in reducing the sliding friction between the tool and workpiece wall, reducing the BUE formation. This also furthered the entrapment of wear debris in dry cutting. However, this effect caused less chip evacuation from the area, preventing chips being taken away from the processing area and chip thickening at feed rates greater than 0.06 mm/rev and 50 m/min. The result showed a clear reduction in the adhesion of titanium on the drill bit but not a reduction in the cutting forces and process temperature. Texturing the drill bit also changed the chip formation in the helical groove giving very little clogging [127]. The secondary cutting edge was concentrated at the upper edges of micro-textures in drill bits. This was avoided by increasing the width of the micro texture. Simulation models often have large errors because of this secondary cutting edge of chips [47].

### 3.4. Lubricants

**Figure 28:**
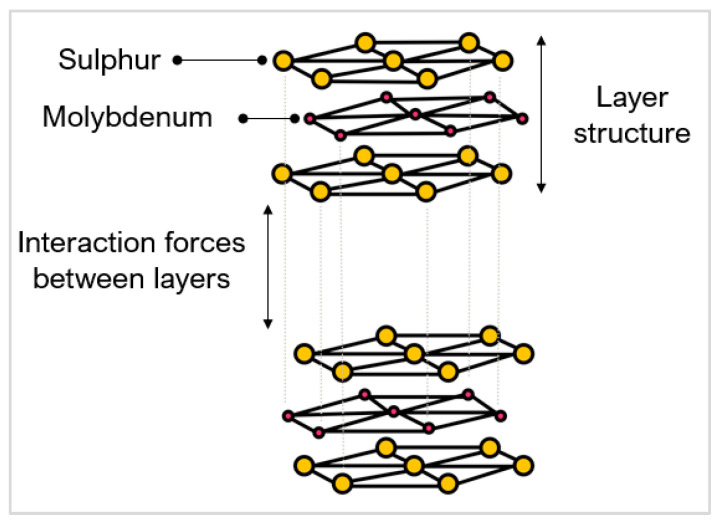
Molybdenum disulphide layer structure.

Textures act as reservoirs for ductile chips and for lubricant retention. Coolants are used to reduce cutting temperatures, avoid poor cutting interaction, and eliminate BUE. They also aid in lubrication to reduce the friction between the tool and the workpiece [172]. The need for improved dry cutting is high as coolants are becoming phased out due to their negative impact on the environment [173,174]. The methods for adding solid lubricants are non-laser based; this includes burnishing [134] and physical vapour deposition (PVD) [175]. Recent work and studies have shown that solid lubricants can have significant improvement in various areas of the cutting process giving a comparable performance to liquid coolants, including better tribological behaviour and preventing adhesion. Solid lubricants have a lamella microstructure with strong covalent bonding within layers but weak van der Waal bonding between the layers [174] (Figure 28). They have the ability to withstand high loads during an operation but can still shear across the surface when a transverse force is applied. The main solid lubricants used with ultra-hard materials are MoS_2_ and tungsten disulphide (WS_2_) to prevent adhesion and enable tribological-chemical reactions [176]. These lubricants have also been the main choice with carbides. MoS_2_ is the most commonly used, but WS_2_ provides a similar performance [105,146].

In textured tools, the use of solid lubricants further improves heat dissipation and lowers the cutting temperature. During an operation, lubricant expulsion from micro-textures creates a smearing effect; this alters the chip interaction and reduces the COF. The smeared layer also helps distribute load forces.

Jianxin et al. [146] created linear grooves on WC-Ti/Co rake face using a femtosecond laser. Table 7 shows the comparison of cutting results with and without the addition of WS_2_ lubrication in the turning of hardened steel at an average speed of 250 m/min. Both the textured and lubricant filled textured with WS_2_ performed better than the untextured tool, but the lubricant filled texture outperformed the unlubricated textured tool when turning hardened steel.

Wu et al. [45] and Deng et al. [105] experiments presented similar performance improvements using MoS_2_ as the Jianxin et al. [146] study with textured WC-Co tools. Gajrani et al. [141] also compared the performance of textured tools with and without MoS_2_ on WC; the cutting forces were reduced by 4.23–10.82% and 7.31–17.41% respectively. Wu et al. [165] studied the tribological behaviour of a linearly textured WC tool via ball-on-disk test on Ti_6_Al_4_V. The use of solid lubricant reduced the size of wear scars on the tool and wear rate of the titanium balls. Zhang et al. [108] corroborated this by studying the tribological performance after texturing WC with different linear patterns. The crater, flank wear, and corner radius wear exhibited by the lubricated tool were lower than the unlubricated tool. Jianxin et al. [132] noticed an increase in abrasive wear with the WS_2_ solid lubricants, when using smaller texture densities on a WC tool. The texture geometry also influences the lubricant behaviour. Though a systematic comparison has not been done yet, current literature shows that dimples textures interact with lubricants best, characterised by a reduced rake and flank wear [42,118,130,176].

One area that is worth considering is the possibility of finding new methods to induce lubricant like properties in the parent material surfaces via laser processing. Allotropical changes have been demonstrated in ultra-hard materials (Section 2.1), most commonly, in PCD by making regions of graphitic region and in PcBN by amorphous BN and hBN. Despite being a by-product of the laser process, hBN acts as a solid lubricant aiding in the cutting performance during application testing. Breidenstein et al. [93] also demonstrated the formation of hBN when laser texturing PcBN. Higher fluences (>3.7 J/cm^2^) increased the extent of hBN. The presence of hBN within the textures reduced the cutting forces when turning Inconel 718. The tool life was prolonged and stabilised the cutting edge. Pacella and Brigginshaw [94] also found this when texturing PcBN; hBN was concentrated in the deepest part of the groove, which corresponded to the highest energy density during laser machining. Functionalising the laser process to produce a controlled amount of hBN in specific regions to act as a solid lubricant would be useful instead of using a separate and distinct manufacturing process to add a lubricant. Controlling the structures in other hard materials groups such as carbides could provide the opportunity to uniquely control the microstructural behaviour of a cutting tools and induce lubricant-like properties in a low cost and accessible way.

## 4. Conclusions

This review investigated the current trends in laser fabrication of surface textures on hard and ultra-hard cutting tool materials (PCD, PcBN, and WC) to improve their cutting performance. The following conclusions were highlighted from this critical review:

- Pulsed laser ablation is an established surface texturing technique, providing a relatively accurate, non-contact, and flexible method for machining difficult to machine materials. Material removal occurs via melting, vaporisation, and sublimation of the processing region. Laser parameters have a direct impact on the physical mechanisms governing the texturing process, the material removal rate, the surface integrity, the microstructure, and the quality on textures generated.
- Processing at near IR wavelengths (1064 nm) can ablate hard and ultra-hard materials; however, the absorptivity and optical breakdown efficiency of laser processing is improved with shorter wavelengths (<532 nm) as these have higher energy pulses. This is ideal for ablation as it creates an efficient medium for molecular bond breaking, limiting thermal conduction to surrounding regions and minimising the size of thermal defects such as HAZ to as small as 1 μm regions.
- Pulse duration significantly dictates the material photon absorption. From the microsecond to the femtosecond regime, the photon absorption progressively changes from linear to multiphoton. Multiphoton absorption increases the energy transfer to the microstructure. Textures processed in the nanosecond range exhibited allotropic transitions, melting, and recrystallisation of the binder phase. Towards the picosecond range, there is a reduced risk of thermally induced defects like melt debris and recast layer, but thermal defects (such as HAZ and phase transitions) were still evident due to the superheating of the processing region. The femtosecond pulse duration resulted in least amount of these defects, leaving smoother and uniform texture profiles.
- Ablation threshold is dependent on the material composition (binder composition, binder percentage, and hard grain size) and fluence. The fluence alters the ablation mechanism with a noticeable shift towards material sublimation from melting as the fluence passes the ablation threshold. At low fluence (close to the ablation threshold), texture surfaces are smoother but shallower, requiring multiple passes to achieve the desired depth and shape, often leading to less precise material removal, cracking, and melt debris. Higher fluences cause deeper features, made in fewer passes, but can be accompanied with an increased surface roughness and distinct boundaries of allotropic transitions.
- A range of surface textures can be produced using lasers; however, the complexity and configuration of textures must be selected carefully to limit prolonged and unnecessary laser processing and avoid defects discussed in this review. Textures are typically made to widths between 50 and 100 μm and depths of 0–100 μm. The majority of studies manufactured linear grooves, dimples, and rectangular pits to shallower depths to minimise thermal damage and maintain the surface integrity of the tool region. The optimisation of laser parameters highlighted can generate an array of uniform surface textures with consistent geometric tolerances and microstructures.
- Laser surface texturing creates of textures in various sizes, geometries, area ratios, and orientations in different locations of a tool. Commonly, the rake face (single point cutting) and the flute (multi-point cutting) are textured as these are regions of initial contact and chip-tool interaction. Texturing these regions alters the contact length and chip flow behaviour; furthermore, the textures act as reservoirs for lubricant and debris. The tribological performance of textured tools was reviewed in this paper, concluding that textures are effective in reducing COF, cutting forces, reducing wear debris, decreasing adhesion, and allowing for better heat dissipation at the cutting interface. The performance of textures is dependent on texture characteristics (texture size, orientation, geometry, area ratio, and density) and conditions of machining (workpiece material, feed speed). Textures that are transverse to the chip flow demonstrate a greater ability to reduce friction and cutting forces because of the reduced contact length.
- The geometry of texture affects the chip formation mechanism: rectangular and linear shapes are more likely to generate smaller and more uniform chips throughout the cutting process. A higher width to depth ratio or area ratio also plays a factor in this, but there are limited studies that fully investigate why. Non-conventional texture geometries have shown promise in improving tool chip interactions. The improvements with hybrid or combination textures were linked to chips progressively moving over different textures which changed the pressure distribution during the cutting process.
- The texture density dictates the texture coverage on the tool; this has a direct impact on cutting force and on the COF because it changes the amount of secondary shear zones. Texture density around 10–20% can stabilise adhesion and BUE but density over 30% increases tool wear and adhesion. This is because the proximity of textures causes additional cutting zones as chips flow, leading to abrasive wear. Adhesion build-up between textures is also greater with higher densities, burying textures at a quicker rate.
- Texture geometry influences the tool behaviour with solid lubricants. A smearing effect is created, helping to lubricate the area and distribute forces. Though a systematic comparison is not available due to limited available studies, dimples and elliptical grooves show the best interaction with lubricants; these shapes make it easier for lubricant to be expelled from the texture and smear lubricant onto the tool surface.
- Cutting conditions (workpiece material, feed speed) have a primary role in the performance of textured tools. With aluminium alloy workpieces, lower cutting speeds cause large amounts of adhesion; this quickly buries the textures making the performance comparable to an untextured tool, whereas titanium alloy workpieces are more likely to cause adhesion at higher speeds. Despite this, textured tools have consistently shown better tribological performance at very high speeds up until a point, beyond which the improvement in COF starts to diminish. This point varies for different tools and workpiece materials; in some cases, this can be as low as 125 m/min (PCD tool and Ti6Al4V workpiece) and as high as 250 m/min (WC-Ti/Co tool and hardened steel workpiece).
- Specific to hard and ultra-hard material composites, the binder is also an influential factor in the wear behaviour of surface textures. Textures on tools with harder binder compositions are less likely to show wear in the form of binder breakdown and binder diffusion.

## 5. Future Trends

**Figure 29:**
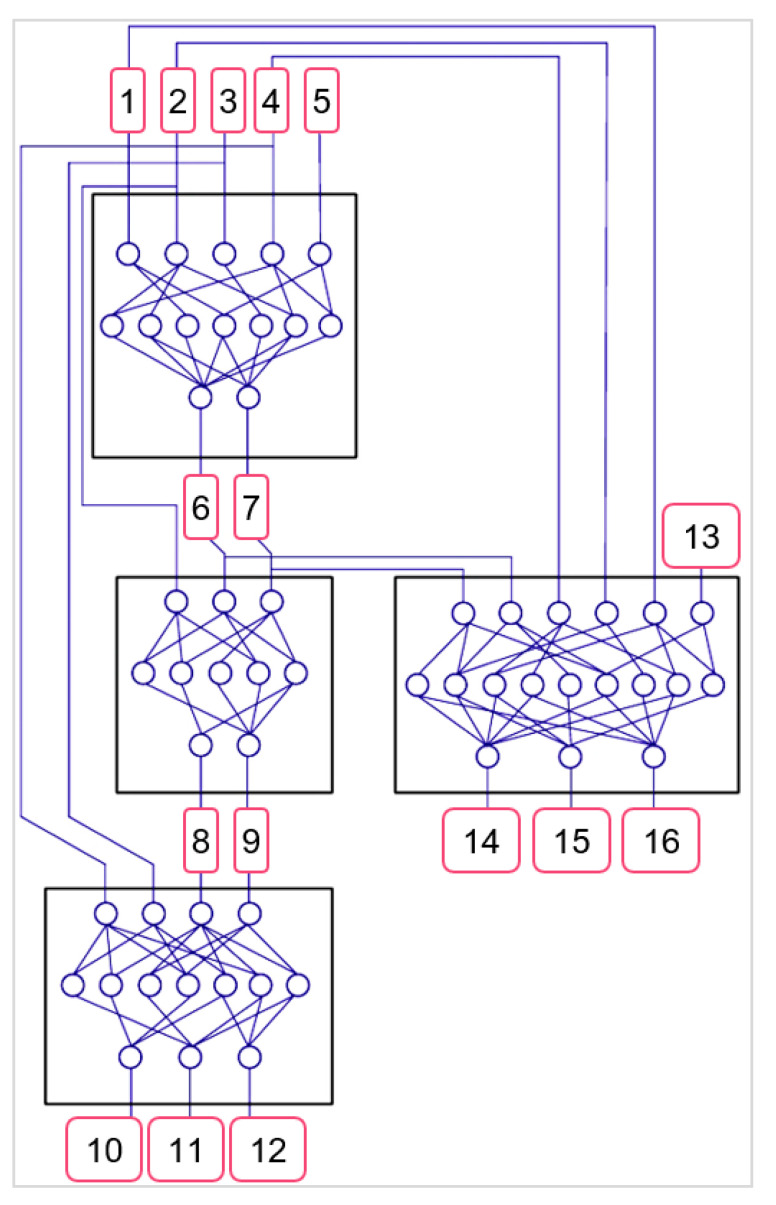
Potential ANN structure to fully describe the laser processing and surface texture performance where 1—Cutting speed, 2—Material property, 3—Surface roughness, 4—Workpiece material, 5—Depth of cut, 6—Texture Density, 7—Area ratio, 8—Width, 9—Depth, 10—Pulse energy, 11 —Laser feed speed, 12—Pulse Duration, 13—Nano-features, 14—Wear, 15—Adhesion, 16—Coefficient of friction.

The critical analysis of laser surface texturing and texture performance of hard and ultra-hard materials in this paper has highlighted key results and conclusions; however, there are still knowledge gaps in recent studies and areas that need to be addressed in future research. The following topics and research areas are suggested to progress the understanding in the field:

- Studies in literature have reported the generation of allotropic transitions as a result of laser processing in PCD and PcBN materials, the extent is dependent on the energy density used during processing. Formation of graphite and hexagonal boron nitride in selected areas of the tool can be used to introduce these allotropes as solid lubricants within the manufacturing process rather than in the material design. Investigating the ability to control the amount of hBN/graphite in specific regions of the textures to customise the tool performance in a low cost and accessible way, could provide an innovative method of inducing lubricant properties to extend the performance of one the most used cutting tool materials.
- The minimum sizes of textures able to produce a noticeable cutting tool performance were 1–2 μm in depth and 1–3 μm in width. Biological structures (shark skin, frog toe pad, snakeskin) have shown that the use of even smaller textures can alter wet/dry adhesion, friction, and strengthen selected regions. Recently, LIPSS have been used to generate surface structures smaller than 1 μm. There is scarce literature on the generation of LIPSS with cutting tool materials in collaboration with micro-textures. The addition of nano-textures within micro-textures could show a similar pattern to nature where significant improved tribological performance is achieved with a slight strengthening effect.
- One interesting area to explore is the use of more hybrid combination and non-uniform textures, for example, varying density of texture based on the tool location. These irregular structures seem to have a greater effect than uniform structures on the type of chip formation mechanism, to control BUE and cutting force stability. It is not clear why this is the case, but fundamental research is needed to understand the cause and inform improvement of the configuration of textures.
- The current rationale of laser parameter selection for the fabrication of surface textures is still based on a trial and error or statical approach. However, the laser/material interaction during laser processing is stochastic and complex. It is difficult to predict the material’s response even employing thermal modelling. Advances in modelling and artificial intelligence are beginning to show their value in understanding the effects of laser parameters, giving users the ability to optimise texture microstructure. From literature, it is not clear if the choice of laser parameters should be dependent on the texture characteristics (e.g., geometry, area ratio, tool location). Using AI would be beneficial in quickly discerning this and tailoring the microstructure to achieve bespoke tribological performance in a range of cutting tool conditions.
- Although there is a clear consensus that the fabrication of micro-textures on cutting tools does improve the cutting performance, the amount of improvement is a multi-factorial decision (texture geometry, texture dimension, density, location, tool material, and machining application). There is a lack of research in the choice of the optimal texture characteristics for a specific machining application to maximise texture performance, promoting the need for advanced modelling. Similar to the recommendation in laser processing, the use of AI and optimisation modelling would be beneficial to create systems that can accurately identify ideal textures and predict the performance of textured tools (wear rate, COF, adhesion). This could allow for the functionalisation of micro-textures for given operational conditions. Figure 29 shows an example proposal with these characteristics in a multi-level ANN structure to demonstrate different dependencies described in this critical review.
- Changes to cutting tool performance are not limited to surface texturing. Studies have shown that any tool edge modification changes chip interaction and the resultant frictional behaviour at the tool-chip interaction. More work is needed in understanding the different types of tool edge modifications (rounding, chamfering, chip breaker designs) possible with laser processing and the potential value in creating these structures for cutting tool performance.
- After use, textures suffer from material shredding, ploughing marks, and texture damage; however, the main structural integrity of the tool is preserved, and there is little indication that there is significant damage beneath texture structures. Research has yet to investigate how to refresh these textures to allow for future use as a cutting tool and further reconditioning methods to extend tool life.

## Figures and Tables

**Table 1:** Hard and ultra-hard hardness categorisation on Vickers hardness scale [3,6].

Metallic Hard Materials >15 GPa	Covalent Hard Materials >15 GPa	Ionic Hard Materials >15 GPa	Ultra-Hard Materials >40 GPa
Tungsten carbide (WC) Titanium nitride (TiN) Titanium carbide (TiC) Chromium nitride (CrN)	Silicon carbide (SiC) Silicon nitride (SiN) Titanium boride (TiB_2_)	Aluminium oxide (Al_2_O_3_) Zirconium oxide (ZrO_2_) Titanium oxide (TiO_2_)	Polycrystalline diamond (PCD) Polycrystalline cubic boron nitride (PcBN) Borocarbonitrides (b_x_C_y_N_z_) Tetraboron carbon (B_4_C) Natural diamond

**Table 3:** Relevant laser and processing parameters associated with surface texturing in cutting tools, included this review.

Laser Parameters	Texture Characteristics	Application Characteristics	Machining Measurements
Wavelength Pulse duration Laser speed Fluence Laser source	Texture geometry Depth Width Width/area ratio Texture density Nano structures	Depth of cut Workpiece material Surface roughness Cutting speed	Coefficient of Friction Adhesion Wear

**Table 4:** Summary of research in pulsed laser ablation of hard and ultra-hard materials.

Ref	Tool	Binder%	Grain Size (μm)	Laser Medium	Wavelength (nm)	Pulse Duration	Spot Size (mm)	Fluence (J/cm^2^)	Feed Speed (mm/s)	Frequency (kHz)	Research Findings
Zhang et al. [58]	PCD	-	25	Nd:YAG KTP/Nd:YAG	1064 532	-	-	-	100, 120	0.05, 10	Regions convert to graphite which sublimates or react with oxygen. High power density cuts via evaporation and sublimation. Low power density cut through melting.
Eberle et al. [59]	PCD	-	-	EWAG	1064	10 ps 125 ns	0.025 0.03	2.12–9.05	-	-	Using 10 picosecond, no residual graphic carbon layer or HAZ is present. Using 125 nanosecond, allotropes of graphitised carbon consisting of low sp3 amorphous carbon are present. Ablation mechanism via ejection of graphitic carbon or optical breakdown avoids graphitisation.
Dumitru et al. [60]	WC, TiC, TiN Diamond TiN	Co, 10 Co, 6	-	Ti:Sapphire	800	100 fs	6	0.13–2.45	-	1.00	Diamonds show the highest ablation threshold. Ablation rate ranges from 0.1–0.2 µm/pulse. WC, TiC, TiN, Diamond, TiN can be processed at low fluences with a high precision. Ablation threshold for WC is greater than 0.1 J/cm^2^.
Pacella et al. [17]	PCD	Co, 15	2	Nd:YAG	1064	20 µs	0.03	848.65	-	100	Abrupt interface graphite in PCD below processing region. Partially evacuated pockets containing traces of Cobalt are found within amorphic/graphitic regions.
Denkena et al. [61]	PcBN	TiCN, 35	3	Nd:YVO4	1064	85 ns	0.04	-	200–800	60	Ablation depth is 30 µm into the surface. There is an increase hardness and compressive residual stress in laser processed regions. Increases of surface roughness on the kerf edge due to microstructure formation.
Denkena et al. [49]	PcBN—A PcBN—B PcBN—C	TiC W-Co-Al AlN	-	-	1064	300 fs 10 ps 90 ns	-	4.68–20.79	-	400, 800, 100	Pulse durations of a few picoseconds reduce the number of phase transformations. Only nanosecond duration shows melting and recrystallisation of the binder material reducing the hardness in these areas.
Okuchi et al. [62]	NPD	-	-	NUV-Nd:YAG Yb:Fibre	1064,355,1045	80 ns, 100 ns 700 fs	0.03 0.002 0.001	3.18	0.05–2.00	-	Nanosecond near-ultraviolet wavelength (NUV) and femtosecond Nd:YAG lasers can be used for precise micromachining and surface finishing. NUV is preferable for thin samples. Femtosecond lasers are better for larger area processing as there is no graphite contamination.
Pacella et al. [63]	PCD	Co, 8 Co, 15	25 2	Nd:YAG	-	10–30 µs	0.04	-	100–900	10–50	Coarse and fine grains have similar reaction to ablation even with different cobalt percentages. Lower speed (increased fluence) causes higher quantities of Cobalt melt and redeposition. Percentage of Cobalt redeposition in ablated areas is proportional to Cobalt extent before ablation. Ablation on a continuous groove on the fine grains shows higher Cobalt percentage than coarse.
Pacella et al. [40]	PCD (CTB010, CTM302)	Co, 12 Co, 10	10, 2–30	Yb:Fibre	1064	220 ns 45 ns	0.04	3.78–11.34	70–350	35, 105	Fluences above 11.34 J/cm^2^ caused changes to milling mechanism. Fluences less than 20 J/cm^2^ resulted in more controlled microstructural changes, leading to better surface integrity. At slower speed thermal energy, metastable diamond is converted to stable graphite. Cobalt melt pool expansion makes compressive stress on diamond grains. Recoil pressure pushes carbon and graphite to sides of grooves and ejected.
Pacella et al. [8]	PcBN	TiC, 50	1.5	Nd:YAG	1064	10–50 µs	0.04	623.00–7369.00	100	10	Allotropic transformation of PcBN to amorphous BN immediately below the ablated surface. hBN is present at depths exceeding 300 nm. Boundaries between BN and binder remain intact. High fluence showed ordered bands of BN allotropic transitions below the surface with the deeper material unaffected.
Butler-Smith et al. [64]	PCD	Co, 7	5	-	1064	12 ps	-	6.70	-	800	Pulse laser ablation had better geometric flexibility compared to electro-discharge-grinding on PCD. There were distinct differences in the substructures at nanoscale.

**Table 5:** Summary of research in laser surface texturing of hard and ultra-hard materials.

Ref	Tool/Workpiece Material	Texture Pattern	Texture Dimension d—Depth μm, w—Width μm, x—Pitch μm	Location	Lubricant	Laser	Key Findings
Breidenstein et al. [93]	PcBN-TiCN for turning Inconel 718	Array of dimples	d = 2 w = 25, 40	Rake face, Flank face	-	Nd:YVO_4_ laser, 1064 nm, 3–4.4 J/cm^2^, 70 ns pulse duration	Laser texturing induces transformation from cBN to hBN. During cutting, the hBN acts as a solid lubricant, decreasing the cutting forces. Wear behaviour links to hexagonal formation with a decreased hardness.
Ghosh and Pacella [100]	PCD-Co cutting tool for turning Al 6082	Parallel grooves to CFD, Perpendicular grooves to CFD, Parallel to MCE	d = 0.26 w = 7 x = 20	Rake face	-	Fibre laser, 1064 nm, with 260 ns pulse duration	Overall microgrooves on rake face reduce cutting forces and improve the frictional behaviour of chips. Aluminium chips adhere strongly to cutting tools. Perpendicular grooves reduce cutting force by 23% and surface quality improves by 11.8%. Parallel grooves reduce force by 11.76% and friction coefficient by 14.28%.
Pacella and Brigginshaw [94]	PcBN-TiN cutting tool for turning AISI51200	Orthogonal grooves, Perpendicular grooves, Linear Crosshatch	d = 1–2 w = 30 x = 75	Rake face	-	Yb:Fibre laser with 46 ns pulse duration	Perpendicular grooves and crosshatch show better flank wear resistance. Perpendicular grooves have purely crater wear on chamfer edge. Crosshatch design has wider but shallower crater wear with localised chipping on flank face from stress concentrations. Laser processing induces solid lubricant hBN which improves heat dissipation.
Xing et al. [103]	TiCN cutting tools for turning Al 6061	Linear grooves Dimples Rectangular pits	d = 10 w = 70–200	Rake face	-	Nd:YVO_4_ laser, 355 nm, with 8 ps pulse duration	Fluctuations of cutting forces are smaller. At low speeds, there are higher forces because of strain hardening. High speeds result in lower forces from thermal softening and drop in shear strength. Rectangular pits have the smallest cutting force and most uniform chips.
Su et al. [92]	PCD cutting tool for turning Ti_6_Al_4_V	Linear grooves	d = 51.5, 55.4, 54.4 w = 60.4, 60.4, 56.5 x = 86.4, 83.9, 84.5	Rake face	-	Fibre laser	Better tribological properties on tool-chip interface from textures. Titanium alloy workpieces adhere significantly to the cutting tool causing rapid wear. Grooves decrease contact length. Formation of TiC from the reaction of the carbon in the PCD. TiC as a by-product is favourable as it protects the surface.
Law et al. [164]	PcBN cutting tool for turning hardened bearing steel	Linear grooves, Dimples	d = 5–20 w = 15–60 x = 70–150	Rake faceFlank face	-	Fibre laser, 1070 nm, 90 ns pulse duration	Textures in various orientations and sizes are fabricated with a laser. Significant crater wear is observed for all cutting tools. Textures in the vicinity of craters hasten the wear. Textures away from craters do not influence the cutting performance. The amount of chip adhesion and chip morphology is relatively independent of texture type.
Jianxin et al. [146]	WC-TiC/Co cutting tool for turning #45 steel	Elliptical grooves		Rake face	WS_2_	Ti laser with 120 fs pulse duration	Elliptical shape is chosen because wear craters on standard cutting tools have an elliptical geometry. Textures filled with WS_2_ have the most improved performance, smallest cutting forces, temperature, and frictional coefficient. Improvements from the reduced contact length and thin self-lubricating layer.
Jianxin et al. [105]	WC-Co cutting tool for turning #45 steel	Elliptical grooves, Parallel Linear, Linear Grooves	d = 200 w = 50	Rake face	MoS_2_	-	Elliptical grooves perform best with a 20% reduction in cutting force. The low shear lubricating film is easily released from grooves and smears onto the rake face. There is a reduced contact length from 1mm to 0.8mm at the tool interface.
Zhang et al. [74]	WC-Co for ball-on-disc test on SAE 1045 steel	Linear grooves (radial around disc)	d = 26, 45, 51, 500 w = 40, 70, 100, 130	-	WS_2_	UV laser, 355 nm	Coefficient of friction reduces from 0.301 to 0.275 in dry cutting and 0.301 to 0.138 with the addition of solid lubricants. Larger area ratio and smaller texture width could diminish the friction coefficient.
Ze et al. [45]	WC-Co for ball-on-flat on Ti_6_Al_4_V	Dimples	d = 100 w = 50 x = 200, 250, 300	Rake face	MoS_2_	Nd:YAG laser, 1064 nm, 20 ns pulse duration	Taguchi method with sliding speed, load, and area density as factors. MoS_2_ reduces the average friction coefficient on the wear track. Area density of textures plays a role in improving tribological performance with a reliability of 90%.
Mishra et al. [109]	WC-Co with AlTiN, AlCrN coatings for turning Ti_6_Al_4_V	Chevron	d = 25	Rake face	-	Nd:YAG laser, 1064 nm, 20 ns pulse	Coatings applied after laser texturing by PVD. Laser ablation causes formation of cobalt oxide which helps the mechanical interlocking improving adhesion. Lower flank wear from the AlCrN coating.
Sugihara and Enonmoto [122]	WC-Co cutting tool for milling medium carbon steel	Linear grooves	d = 5 w = 20 x = 20	Rake face Flank face	-	Ti:Sapphire laser, 800 nm, 150 fs pulse duration	Micro groove texture on rake face limits crater wear as they act as reservoirs and traps for debris. Optimum texture is dependent on the cutting conditions (e.g., speed, wet or dry). Different wear mechanism when texture was on flank face.
Ze et al. [165]	WC-Co for ball-on-disk test	Linear grooves	d = 100 w = 50	Surface	MoS_2_	Nd:YAG laser, 1064 nm, 20 ns pulse	An untextured surface with lubricant performs better than textured surface without lubricant. Textures with lubricants perform best, reducing frictional coefficient by 20–25% and temperatures by 8–15%.
Martinez-Vazquez et al. [95]	WC for turning on Ti_6_Al_4_V	Linear grooves, Dimples, Circular grooves	w = 100–150	Surface	-	Yb:Fibre laser, 1070 nm, 100 ns	Linear grooves improve the lubricant retention of the surface. Dimples help conduct cutting fluid to the cutting tool face interaction. Different texture geometries modify the lubricant retention.
Niketh and Samuel [127]	WC drilling tool for Ti_6_Al_4_V	Dimples Linear grooves	d = 60, 40 w = 90, 50 x = 50, 80	Flute Margin	-	Nd:YAG laser, 1064 nm, 20 ns pulse	Textures show a 10.68% reduction in force and 12.33% reduction in torque. Less clogging of chips in the flute texture. Chip formation changed during various stages of machining. Micro dimples effective in reducing sliding friction between the drill tool and machined hole wall surface, so lessened the BUE formation.
Liang et al. [56]	WC-Ni_3_Al/Co for Reciprocating sliding test Ti_6_Al_4_V	Linear grooves	d = 100, 110 x = 150	Surface	-	Fibre laser, 1064 nm, 100 ns pulse duration	Wear resistance in Wo-Ni_3_Al binder was superior to WC-Co in terms of friction coefficient, wear loss and characteristics of worn surface. Sandpaper removes bulges from the result and ultrasonically cleaned in fresh dehydrated alcohol. Wear starts from initial chipping of micro-groove edge and then propagates to convex region—the space between grooves. Hardness in micro-groove edge decreases after laser for both binders, but less for Wo-Ni_3_Al.
Pacella et al. [73]	PCD and PcBN for abrasion of SiO_2_	Micro-array	w = 100–250	Abrasive face	-	Nd:YAG laser, 1064 nm, 10 µs pulse duration	Fine grain structures show better wear resistance and lower contact pressures. Coarse grain structures show better shock resistance. Less thermal mismatch meant cracks took 33% longer to present.
Kummel et al. [125]	WC-Co for turning SAE 1045 steel	Dimples, Parallel grooves, Perpendicular grooves	d = 20, 20 w = 50, 50	Rake face	-	Yb:Fibre laser, 1040 nm	Low velocities generated a larger BUE layer. BUE is unstable and deteriorated the workpiece surface. Dimple texture helps stabilise BUE with better wear behaviour on the corner radius wear. Grooves destabilise BUE while the wear of cutting tool increases. Dimples show the smallest wear progression especially at corner radius. BUE is also stabilised. SEM of dimple surface shows better mechanical interlock between BUE and micro-textures.

**Table 6:** Texture performance compared to untextured tool when turning a pure iron workpiece at 120 m/min, negative value implies reduction [110].

Cutting Parameter	Grooves (GT)	Dimples (PT)	Hybrid Texture (GPT)
**Axial thurst force (Fx)**	−4.9%	−22.0%	−39.0%
**Radial thrust force (Fy)**	−6.9%	−20.5%	−30.8%
**Main cutting force (Fz)**	−2.3%	−8.6%	−12.7%
**Temperature**	−7.4%	−21.0%	−28.4%
**COF**	−4.7%	−11.8%	−18.8%

**Table 7:** Comparison of textured tool with and without WS_2_ solid lubrication [146].

Cutting Parameter	Textured Surface	Textured Surface Filled with WS_2_
**Cutting force**	Reduced by 13–22%	Reduced by 25–44%
**Cutting temperature**	Reduced by 9%	Reduced by 16%
**Coefficient of Friction**	-	Reduced by 13–26%
**Wear**	Grooves buried by adhesion, but textures remain visible.	No mechanical ploughing or adhered materials. Filled textures visible.
**Flank wear**	Good resistance	Good resistance

## Data Availability

No new data were created or analysed in this study. Data sharing is not applicable to this article.
